# SPINK1 as a plasma marker for tumor hypoxia and a therapeutic target for radiosensitization

**DOI:** 10.1172/jci.insight.148135

**Published:** 2021-11-08

**Authors:** Tatsuya Suwa, Minoru Kobayashi, Yukari Shirai, Jin-Min Nam, Yoshiaki Tabuchi, Norihiko Takeda, Shusuke Akamatsu, Osamu Ogawa, Takashi Mizowaki, Ester M. Hammond, Hiroshi Harada

**Affiliations:** 1 Laboratory of Cancer Cell Biology and; 2 Department of Genome Dynamics, Radiation Biology Center, Graduate School of Biostudies, Kyoto University, Kyoto, Japan.; 3 Department of Radiation Oncology and Image-applied Therapy, Graduate School of Medicine, Kyoto University, Kyoto, Japan.; 4 Division of Molecular Genetics Research, Life Science Research Center, University of Toyama, Toyama, Japan.; 5 Division of Cardiology and Metabolism, Center for Molecular Medicine, Jichi Medical University, Tochigi, Japan.; 6 Department of Urology, Graduate School of Medicine, Kyoto University, Kyoto, Japan.; 7 MRC Oxford Institute for Radiation Oncology, Department of Oncology, University of Oxford, Oxford, United Kingdom.

**Keywords:** Cell Biology, Oncology, Cancer, Hypoxia, Radiation therapy

## Abstract

Hypoxia is associated with tumor radioresistance; therefore, a predictive marker for tumor hypoxia and a rational target to overcome it have been sought to realize personalized radiotherapy. Here, we show that serine protease inhibitor Kazal type I (SPINK1) meets these 2 criteria. SPINK1 expression was induced upon hypoxia (O_2_ < 0.1%) at the transcription initiation level in a HIF-dependent manner, causing an increase in secreted SPINK1 levels. SPINK1 proteins were detected both within and around hypoxic regions of xenografted and clinical tumor tissues, and their plasma levels increased in response to decreased oxygen supply to xenografts. Secreted SPINK1 proteins enhanced radioresistance of cancer cells even under normoxic conditions in EGFR-dependent and nuclear factor erythroid 2–related factor 2–dependent (Nrf2-dependent) manners and accelerated tumor growth after radiotherapy. An anti-SPINK1 neutralizing antibody exhibited a radiosensitizing effect. These results suggest that SPINK1 secreted from hypoxic cells protects the surrounding and relatively oxygenated cancer cells from radiation in a paracrine manner, justifying the use of SPINK1 as a target for radiosensitization and a plasma marker for predicting tumor hypoxia.

## Graphical abstract

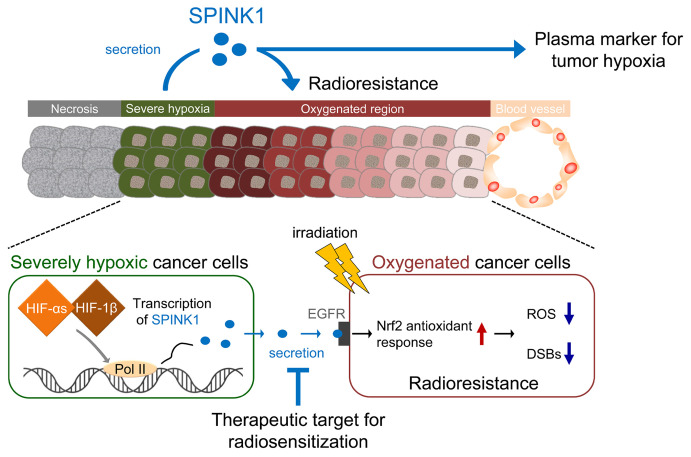

## Introduction

Heterogeneity of tumor oxygenation, which results from imbalances between oxygen supply from the tumor vasculature and the oxygen demand of cancer cells and between the rate of vascular network development and that of cancer cell proliferation, is a characteristic feature of solid tumors (1, 2). Accumulating evidence has shown that tumor hypoxia is strongly associated with many aspects of tumor malignant phenotypes. Cancer radioresistance is one of them; cancer cells are approximately 3-fold more radioresistant under hypoxia compared with normoxia due to both radiochemical and radiobiological mechanisms (3), ultimately having a negative effect on the outcome of radiation therapy (4–7).

To overcome the hypoxia-induced radioresistance, many kinds of hypoxia-targeting strategies have been attempted (2), for example, delivery of molecular oxygen to hypoxic regions, combination with hypoxic cell radiosensitizers, and dose escalation to hypoxic tumor cells using intensity-modulated radiotherapy. Their effectiveness for local tumor control and/or overall survival is confirmed in a clinical setting as well as preclinical studies to varying degrees (2, 6, 8, 9). However, none of these strategies has yet to be applied for clinical use with satisfactory results, which is, at least in part, attributed to limited understanding of the molecular pathways that determine hypoxic cell radioresistance.

Basic, translational, and clinical studies all support the conclusion that HIF-1 is associated with radioresistance of hypoxic tumor cells. HIF-1, which is composed of HIF-1α and HIF-1β, is responsible for the expression of hundreds of hypoxia-responsive genes and, therefore, is recognized as a master transcription factor for the adaptive response to hypoxia. Multiple mechanisms have been elucidated to explain the function of HIF-1 and its isoforms (hereinafter collectively referred to as HIFs; refs. 2, 10–13); however, it is also suggested that mechanisms underlying HIF-mediated radioresistance have not been fully elucidated.

Clinical studies conducted in a variety of cancer types have concluded that the size of the hypoxic fraction varies from tumor to tumor and that a high hypoxic fraction is predictive of a poor outcome after radiotherapy. This finding underpins the motivation to develop a strategy for evaluating tumor hypoxia. The partial oxygen pressure (pO_2_) in a tumor tissue can be directly measured with pO_2_ histography; however, this is not in widespread clinical use because it is highly invasive (14, 15). IHC for intrinsic hypoxia markers such as regulatory subunit of HIF-1, HIF-1α, and its downstream genes can also be used to assess tumor hypoxia (16–18); however, when applied for a small clinical biopsy sample, it may not reflect the hypoxic fraction of the entire tumor. Although PET-based molecular imaging has the advantage of being noninvasive and offers monitoring in real time (19, 20), the radionuclides, in general, cannot be applied to the same patient multiple times. Therefore, an alternative method free from the above-mentioned disadvantages is sought with the expectation of realizing personalized cancer therapy (6).

Because of the limited distance molecular oxygen diffuses through metabolically active tumor tissue from blood vessels, an oxygen gradient develops according to the distance from vessels and leads to a heterogeneous oxygen tumor microenvironment. Cancer cells can be categorized into 3 layers based on the distance from a tumor blood vessel: a well-oxygenated layer, a mildly hypoxic layer, and a severely hypoxic layer. Our previous IHC analysis clearly demonstrated that ionizing radiation causes significantly less DNA damage in both severely and mildly hypoxic layers compared with well-oxygenated layers (21). It seems reasonable to consider that the characteristics of mildly hypoxic cancer cells are attributed to the radiochemical mechanism because of their lower pO_2_ compared with normoxic layers. However, we cannot exclude the possibility that they are caused by an unknown factor secreted from severely hypoxic cells. If this is the case, and if such a factor is secreted into plasma, then the factor could be a convenient marker to predict the hypoxic burden as well as a rational target for radiosensitization.

In the present study, we identified serine peptidase inhibitor Kazal type 1 (SPINK1) as a protein that meets the following 2 criteria: it secreted from hypoxic cancer cells into plasma and induced radioresistance of cancers. Here, we confirmed its usefulness as a plasma marker to evaluate tumor hypoxia and as a potential therapeutic target for hypoxic cell radiosensitization.

## Results

### Identification of SPINK1 as a candidate plasma marker for tumor hypoxia and therapeutic target for radiosensitization.

**Figure 1:**
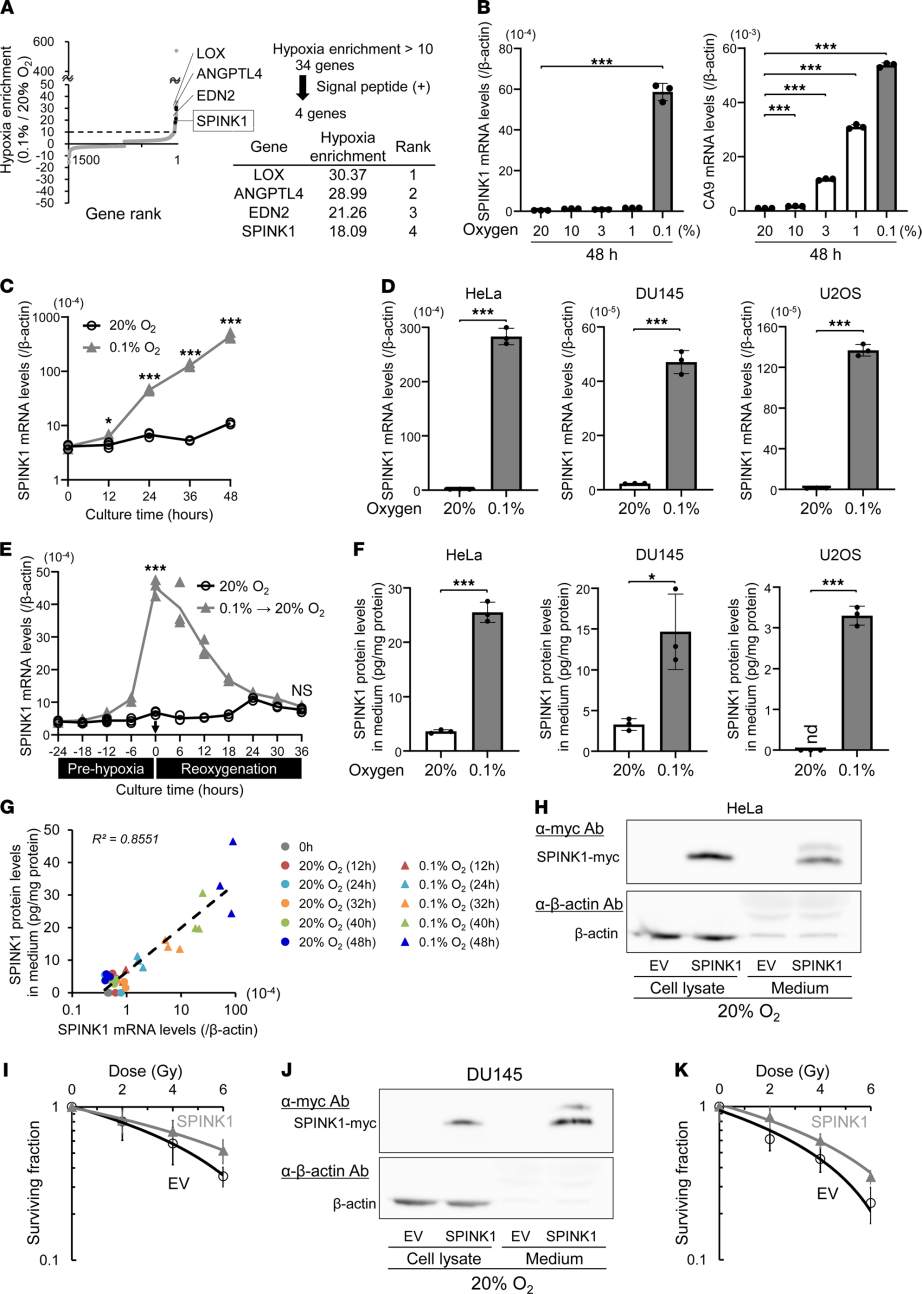
SPINK1 is identified as a candidate plasma marker for tumor hypoxia and potential therapeutic target for radiosensitization. (**A**) HeLa cells were cultured under the indicated oxygen conditions for 24 hours and subjected to DNA microarray analysis. Of 34 genes that exhibited more than 10-fold induction upon hypoxia, the top 4 genes harboring the N-terminus signal peptide are listed. (**B **and** C**) HeLa cells were cultured under the indicated oxygen conditions for the indicated periods, and subjected to qPCR for the indicated genes. (**D**) After being cultured under the indicated oxygen conditions for 48 hours, cell lysates were subjected to qPCR. (**E**) Changes in the SPINK1 mRNA levels in HeLa cells were quantified at the indicated time points during (prehypoxia) and after (reoxygenation) the severe hypoxic treatment and represented as mean ± SD. (**F**) After the same treatment as in **D**, culture media were subjected to the ELISA assay. (**G**) Scatter plot for correlation analysis between SPINK1 mRNA levels and secreted SPINK1 protein levels in cells cultured under the indicated oxygen conditions for the indicated periods. (**H**–**K**) The indicated cells were transfected with either pcDNA4/SPINK1 (SPINK1) or its EV and cultured for 48 hours. Then, both culture media and cell lysates were subjected to Western blotting using the indicated antibodies (**H** and **J**), and then, cells were irradiated with the indicated doses of γ-rays and subjected to the clonogenic survival assay (**I** and **K**). The exogenously expressed SPINK1 was detected using anti-myc tag Ab (**H** and **J**). Data are represented as mean ± SD (**B**, **D**, **F**, **I**, and **K**; *n* = 3 in **B**–**G**, *n* = 6 in **I** and **K**). Two-tailed Student’s *t* test. *P < 0.05, ***P < 0.001. SPINK1, serine peptidase inhibitor Kazal type 1; EV, empty vector.

To identify a candidate protein that can be utilized as a predictive plasma marker for tumor hypoxia and therapeutic target for radiosensitization, we first performed DNA microarray analysis and identified hypoxia-responsive genes on a genome-wide scale. The microarray data set was deposited in the NCBI’s Gene Expression Omnibus database (GEO GSE161393). We found that mRNA levels of 34 genes exhibited a more than 10-fold induction in HeLa cells (cervical cancer) upon hypoxic treatment (< 0.1% O_2_ for 24 hours; Figure 1A). Sequence analysis revealed that 4 of the 34 genes harbored a signal peptide for secretion in each of their N-terminus regions. Therefore, we hypothesized that they would be secreted from cells after hypoxia-dependent expression. Of the 4 genes, we decided to focus on SPINK1 because it has been reported to function as a ligand of EGFR (22, 23), and because it was expected to affect cancer radioresistance.

To verify that SPINK1 mRNA expression was induced upon hypoxia, we exposed HeLa cells to various oxygen conditions and carried out quantitative PCR (qPCR) analyses. As expected, the mRNA expression of a representative HIF-1–regulated gene, carbonic anhydrase 9 (CA9), was found to be induced under both mild (1%–10% O_2_) and severe (< 0.1% O_2_) hypoxia. On the other hand, SPINK1 mRNA increased only under more severe conditions (< 0.1% O_2_; Figure 1, B and C). This significant induction in response to severe hypoxia was observed in a variety of cancer cell lines, e.g., a human prostate carcinoma cell line, DU145, and a human osteosarcoma cell line, U2OS, as well (Figure 1D and Supplemental Figure 1; supplemental material available online with this article; https://doi.org/10.1172/jci.insight.148135DS1). To analyze whether SPINK1 mRNA levels reflect reoxygenation, we subsequently analyzed the decay of SPINK1 mRNA after reoxygenation treatment. Sequential qPCR experiments at 6-hour intervals revealed that SPINK1 mRNA, which accumulated during prehypoxic treatment, immediately and markedly started to decrease after reoxygenation (Figure 1E). Next, we examined whether SPINK1 proteins were secreted into the culture medium upon hypoxic treatment. The ELISA assay demonstrated that the amount of secreted SPINK1 protein in the culture medium significantly increased under severe hypoxic conditions in various cancer cell lines, as expected (Figure 1F). Moreover, simultaneous analysis of intracellular SPINK1 mRNA levels and secreted SPINK1 protein levels confirmed that both of them accumulated as the duration of hypoxic treatment increased and that they were positively correlated with each other (Figure 1G; *R^2^* = 0.8551).

To test the potential of SPINK1 to increase radioresistance, we carried out a clonogenic cell survival assay in vitro. Overexpression of SPINK1, which was confirmed to increase SPINK1 protein secreted into the culture medium by Western blotting, significantly induced radioresistance of both HeLa and DU145 cells (Figure 1, H–K, and Table 1 [*P* = 0.0180 and *P* = 0.0307], respectively). These results suggest that SPINK1 would be a candidate protein as a predictive plasma marker for tumor hypoxia and a potential therapeutic target for radiosensitization because of its property to increase radioresistance.

### SPINK1 expression was upregulated at the transcription initiation level under hypoxia in a HIF-dependent manner.

**Figure 2:**
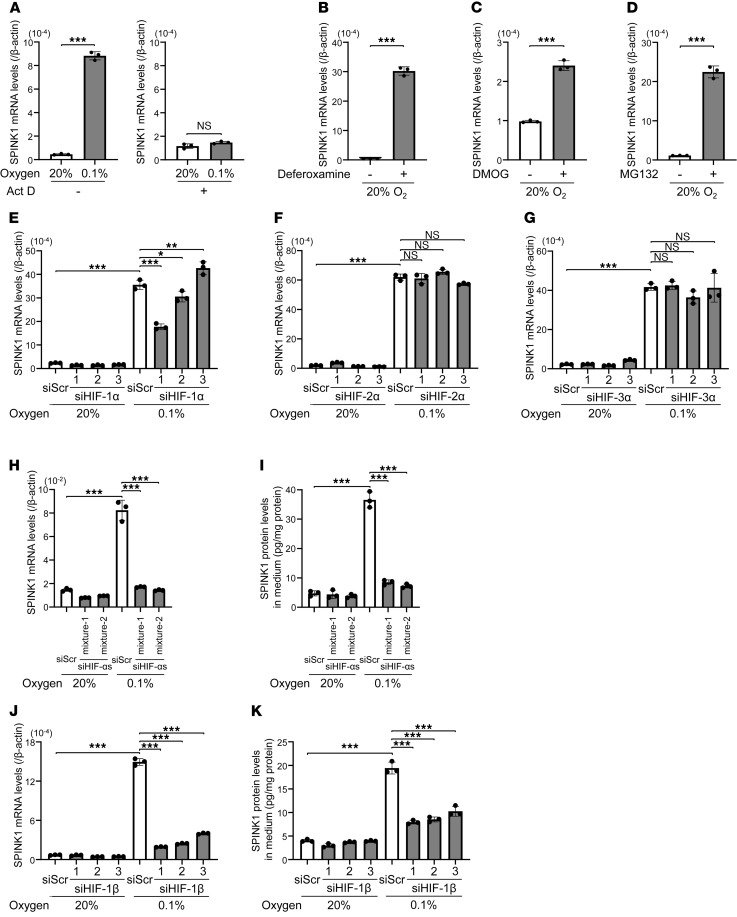
Transcription of the SPINK1 gene is upregulated under hypoxia in a HIF-dependent manner. (**A**–**D**) HeLa cells were cultured under the indicated oxygen conditions in the presence or absence of 5 μg/mL Act D for 24 hours (**A**), 100 μM deferoxamine for 48 hours (**B**), 2 mM DMOG for 24 hours (**C**), or 3 μM MG132 for 12 hours (**D**) and subjected to qPCR. (**E**–**G**) HeLa cells were transfected with the indicated siRNA or scramble siRNA for negative control, cultured under the indicated oxygen conditions for 24 hours, and subjected to qPCR. (**H** and **I**) After simultaneously silencing HIF-1α, HIF-2α, and HIF-3α using 2 kinds of mixtures using HIF-αs and siRNAs (mixture-1 and mixture-2), HeLa cells were cultured under the indicated oxygen conditions for 24 hours and subjected to qPCR (**H**) or the ELISA assay (**I**). (**J** and **K**) The same experiments as in **H** and **I** were conducted after silencing HIF-1β. Scramble siRNA was used as a negative control. Data are represented as mean ± SD (*n* = 3). Two-tailed Student’s *t* test (**A**–**D**). One-way ANOVA with Dunnett’s test (**E**–**K**). *P < 0.05, **P < 0.01, ***P < 0.001. SPINK1, serine peptidase inhibitor Kazal type 1; EV, empty vector; Act D, actinomycin D; DMOG, dimethyloxallyl glycine.

We investigated molecular mechanisms underlying the upregulation of SPINK1 mRNA levels under severe hypoxia. First, we tried to narrow down a critical regulatory step for the induction using a transcription inhibitor, actinomycin D (Act D). When we looked at VEGFA, whose expression is known to be regulated at the transcription initiation level, Act D treatment almost completely suppressed the hypoxia-dependent increase in VEGFA mRNA levels, suggesting that Act D treatment successfully inhibited global transcription (Supplemental Figure 2). When we applied the same Act D treatment to the analysis of SPINK1, the induction of SPINK1 mRNA expression was also inhibited even upon the severe hypoxic treatment (Figure 2A), suggesting that SPINK1 mRNA levels were upregulated at the transcription initiation level under severe hypoxia.

Because HIFs are recognized as master transcription factors for the expression of a wide range of hypoxia-responsive genes, and because there are consensus sequences recognized by HIF-1, which is designated as a hypoxia-response element, in the SPINK1 gene locus, we next examined the possibility that the transcription of the SPINK1 gene is also under the control of HIFs. It has been well established that the activity of HIFs is mainly dependent on the stability of HIF-α proteins and that the stability is sequentially regulated through O_2_/Fe^2+^/α-ketoglutarate–dependent (αKG-dependent) prolyl-hydroxylation by prolyl-4-hydroxylases (PHDs), ubiquitination by von Hippel-Lindau–containing (VHL-containing) E3 ubiquitin ligase, and proteolysis by the 26S proteasome. Therefore, we hypothesized that SPINK1 mRNA expression might be induced by inhibitors of these negative regulators even under normoxic conditions. qPCR consistently demonstrated that an iron chelator, deferoxamine, and an αKG analogue, dimethyloxallyl glycine (DMOG), both of which function as PHD inhibitors, significantly upregulated SPINK1 mRNA levels even under normoxic conditions (Figure 2, B and C). Moreover, SPINK1 mRNA levels were also upregulated by a proteasome inhibitor, MG132 (Figure 2D).

To directly test the involvement of HIFs, we performed qPCR after silencing each of them (Supplemental Figure 3). Silencing of HIF-1α expression by using 3 kinds of siRNAs did not demonstrate a clear tendency that HIF-1 is the sole factor responsible for the hypoxia-dependent expression of SPINK1 mRNA; specifically, 2 of the siRNAs partially canceled the induction, but another enhanced it (Figure 2E). Loss of function studies for the isoform of HIF-2α or HIF-3α showed that they were not associated with the regulation of SPINK1 expression (Figure 2, F and G). On the other hand, the simultaneous silencing of every HIF-α (HIF-1α, HIF-2α, and HIF-3α) almost completely abrogated the hypoxia-dependent increase in the levels of SPINK1 mRNA and secreted SPINK1 protein (Figure 2, H and I, and Supplemental Figure 4). Consistent with these results, silencing the binding partner of HIF-αs, HIF-1β (ARNT; Supplemental Figure 5), canceled the induction of SPINK1 mRNA expression and the increase in secreted SPINK1 protein upon hypoxia (Figure 2, J and K). Taken together, all of these results collectively indicate that the transcription of the SPINK1 gene was upregulated upon severe hypoxia (< 0.1% O_2_) at the transcriptional level in a HIF-dependent manner and that the functions of HIF-1α, HIF-2α, and HIF-3α were more likely to compensate for each other in SPINK1 expression.

### SPINK1 secreted from hypoxic cells induced cancer radioresistance in a paracrine manner and accelerated tumor growth after radiation therapy.

**Figure 3:**
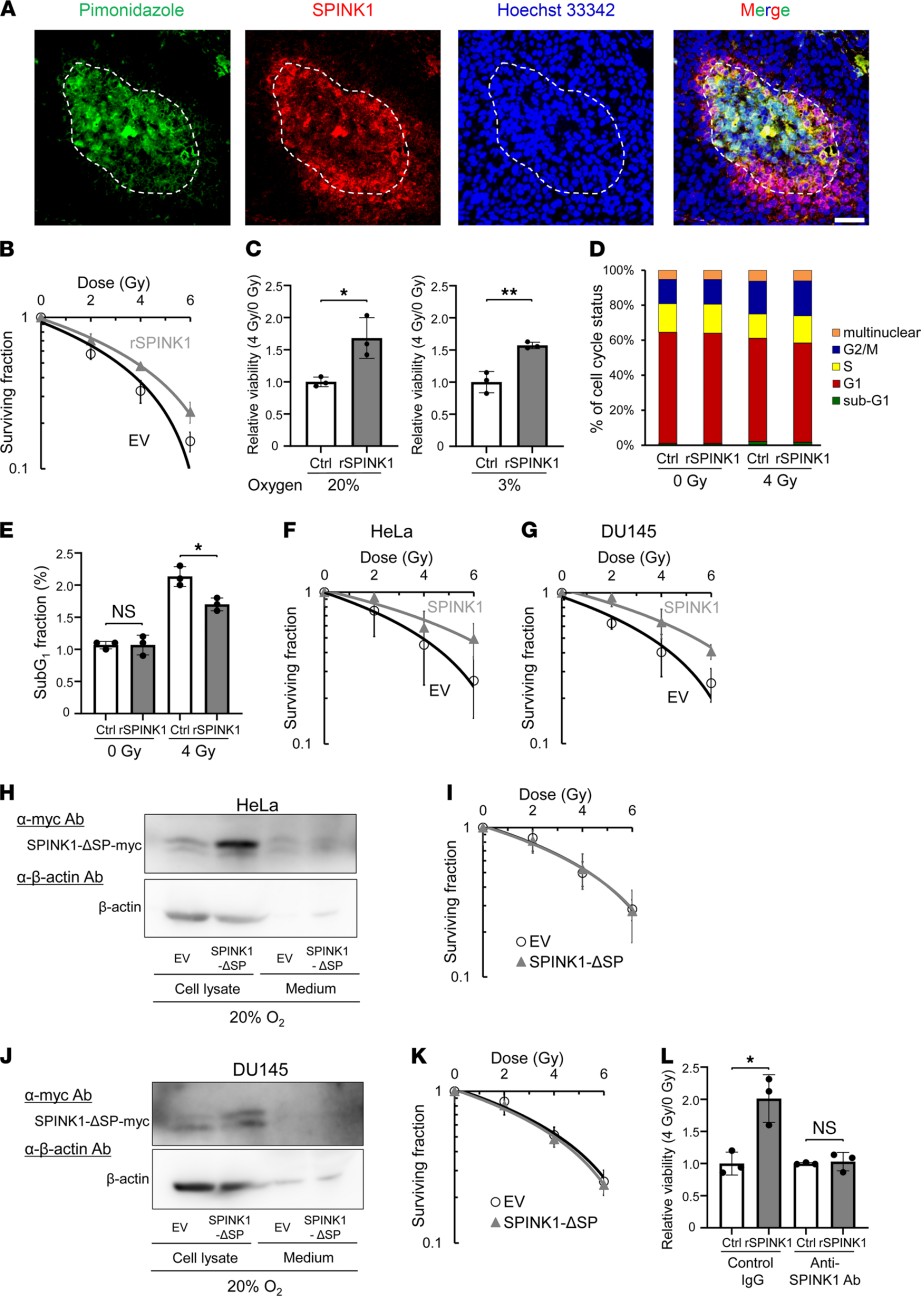
SPINK1 secreted from cells induces cancer radioresistance in a paracrine manner. (**A**) HeLa tumor xenografts were stained with antibodies against a hypoxia marker, pimonidazole (green), or SPINK1 (red). Blue, counter staining with Hoechst 33342. The dotted line represents the outside edge of the pimonidazole-positive regions. Scale bar: 50 μm. (**B**–**E**) After 24 hours serum starvation, DU145 cells were precultured in the presence or absence of 100 ng/mL rSPINK1 for 24 hours, treated with the indicated dose of γ-ray irradiation, and subjected to the clonogenic survival assay (**B**), colorimetric cell viability assay (**C**), and FACS analysis for the cell cycle status (**D**) and sub-G_1_ fraction (**E**). The cells were precultured and irradiated under the indicated oxygen conditions in **C**. (**F** and **G**) After transfection with either pcDNA4/SPINK1 (SPINK1) or its EV, the indicated cells were precultured under mild hypoxic conditions (O_2_ = 3%) for 48 hours, treated with the indicated dose of γ-ray irradiation under the same oxygen conditions as the preculture, and subjected to the clonogenic survival assay. (**H**–**K**) The indicated cells were transfected with either pcDNA4/SPINK1-ΔSP (SPINK1-ΔSP) or its EV and cultured for 48 hours. Then, both the culture media and cell lysates were subjected to Western blotting using the indicated antibodies (**H** and **J**), and then, cells were irradiated with the indicated doses of γ-rays and subjected to the clonogenic survival assay (**I** and **K**). The exogenously expressed SPINK1-ΔSP was detected using anti-myc tag Ab (**H** and **J**). (**L**) After 24 hours serum starvation, DU145 cells were treated with or without 100 ng/mL rSPINK1 in combination with SPINK1-neutralizing antibody or control IgG (0.5 μg/mL) for 24 hours and subjected to the colorimetric cell viability assay. Data are represented as mean ± SD (*n* = 3 in **C**–**E** and **L**, and *n* = 6 in **B**, **F**, **G**, **I**, and **K**). Student’s *t* test. *P < 0.05, **P < 0.01. SPINK1, serine peptidase inhibitor Kazal type 1; EV, empty vector.

IHC analysis of HeLa xenografted tumors using a hypoxia marker, pimonidazole, and an anti-SPINK1 antibody detected SPINK1 protein predominantly in the hypoxic regions and, to some extent, in their surroundings, indicating that SPINK1 protein was secreted from hypoxic regions to relatively oxygenated regions (Figure 3A). To assess whether SPINK1 proteins secreted from hypoxic cells induce radioresistance of oxygenated cells in a paracrine manner, we next performed clonogenic cell survival assays using recombinant SPINK1 protein (rSPINK1; Figure 3B). The surviving fraction showed that rSPINK1 protein significantly increased clonogenic survival after γ-irradiation under oxygenated conditions, such as in the presence of 20% O_2_ (Figure 3B). The dose of radiation needed to kill 50% of cells (D_50_ value) was increased by the rSPINK1 treatment from 3.33 ± 0.21 to 4.10 ± 0.33 Gy, suggesting that SPINK1 induced radioresistance of oxygenated cells in a paracrine manner (Figure 3B and Table 1). A colorimetric cell viability assay also confirmed the potential of rSPINK1 to induce radioresistance under not only 20% O_2_ conditions but also 3% O_2_ conditions (Figure 3C). The increase in the radioresistance was considered to be, at least in part, caused by antiapoptotic activity of SPINK1 but not by the influence of SPINK1 on the cell cycle distribution (Figure 3, D and E). Consistent with cell viability assay, clonogenic cell survival assays also demonstrated that forced expression of SPINK1 caused radioresistance of cancer cells under not only 20% O_2_ conditions (Figure 1, I and K) but also 3% O_2_ conditions (Figure 3, F and G). Although ectopic expression of WT SPINK1 significantly induced radioresistance of cells (Figure 1, H–K), that of mutant SPINK1, which lacked the signal peptide for secretion (herein pcDNA4/SPINK1-ΔSP), did not (Figure 3, H–K, and Table 1). The same results as in Figure 1, H–K, and Figure 3, H–K, were confirmed using SPINK1 knockout DU145 cells: the forced expression of SPINK1, but not that of SPINK1-ΔSP, induced radioresistance of cells (Supplemental Figure 6 and Supplemental Table 4). Moreover, the SPINK1-mediated cancer radioresistance was completely abrogated by a SPINK1-neutralizing antibody (Figure 3L). When clonogenic cell survival assays were conducted under the severe hypoxic condition (O_2_ < 0.1%), overexpression of WT SPINK1 did not enhance radioresistance in our experimental setting (Supplemental Figure 7 and Supplemental Table 4), probably because severe hypoxic treatment induced both expression of endogenous SPINK1 and radioresistance through radiochemical mechanisms, and masked the effect of SPINK1 overexpression. This interpretation was supported by the results of our colorimetric cell viability assays and clonogenic cell survival assays, in which both rSPINK1 and overexpression of SPINK1 induced radioresistance of cells under mild hypoxia at 3% O_2_, whereas SPINK1 expression was not induced at all (Figure 1B and Figure 3, C, F, and G). Together, these data support our hypothesis that SPINK1 protein secreted from hypoxic cells had the potential to cause radioresistance of nearby oxygenated cancer cells in a paracrine manner.

**Figure 4:**
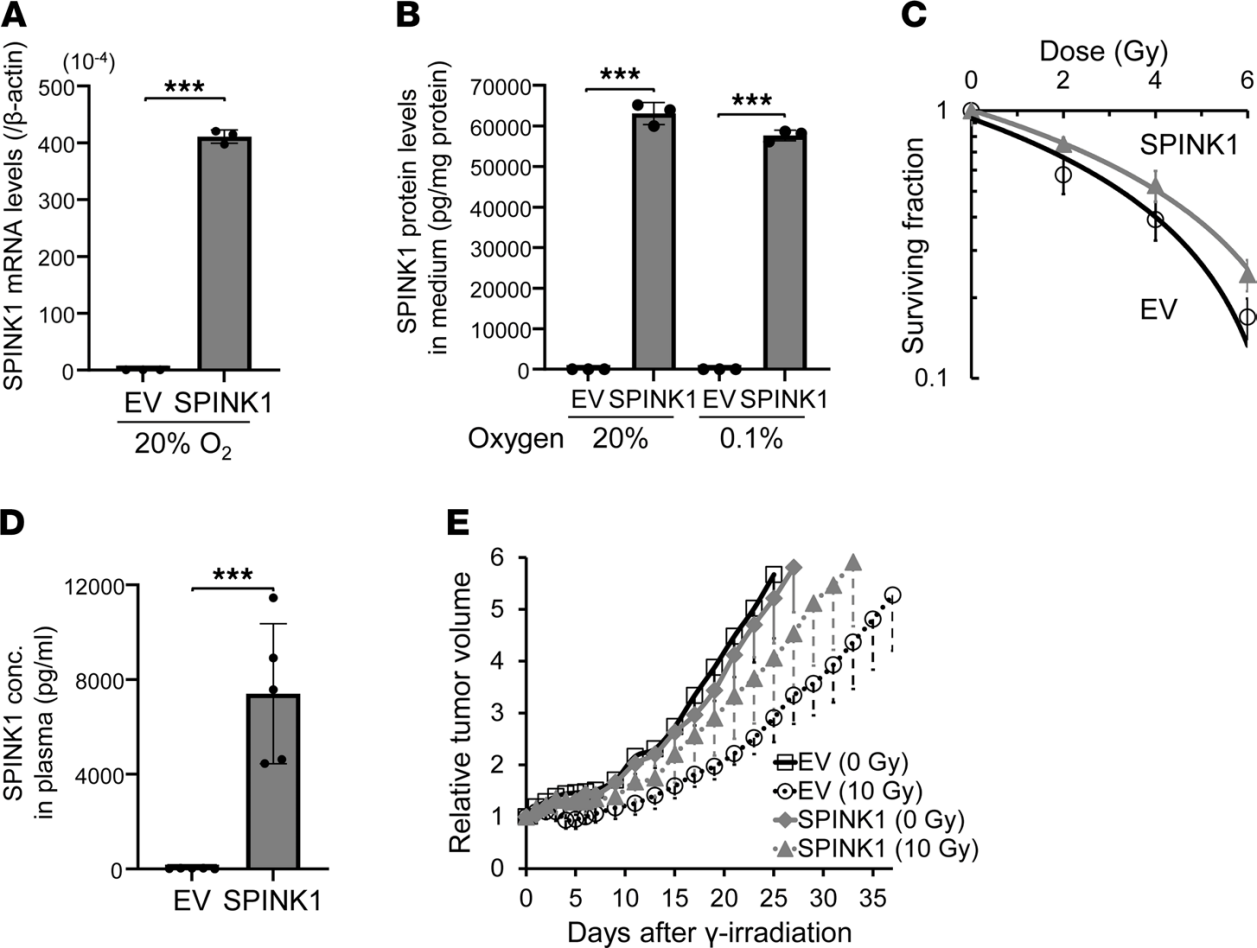
SPINK1 accelerates tumor growth after radiotherapy. (**A**–**C**) DU145/EV and DU145/SPINK1 cells were cultured under the indicated oxygen conditions for 48 hours and subjected to qPCR (**A**) and the ELISA assay (**B**) or treated with the indicated dose of γ-ray irradiation for the clonogenic survival assay (**C**). (**D** and **E**) DU145/EV or SPINK1 xenografts were locally irradiated at a dose of 0 (solid lines) or 10 (dotted lines) Gy. When the volumes of the xenografts reached the same sizes as those on day 0, plasma SPINK1 levels were quantified by ELISA assays (**D**). Tumor growth was analyzed after the treatment (**E**). Data are represented as mean ± SD (*n* = 3 in **A** and **B**, *n* = 6 in **C**, *n* = 5 in **D**, and *n* = 9–10 in **E**). Two-tailed Student’s *t* test. ***P < 0.001. SPINK1, serine peptidase inhibitor Kazal type 1; EV, empty vector.

Next, we examined whether SPINK1 causes tumor radioresistance not only in vitro but also in vivo, using a xenografted tumor model. We established a stable transfectant of DU145 cells with the SPINK1 expression vector (DU145/SPINK1). We confirmed that the cells expressed increased levels of SPINK1 mRNA (Figure 4A) and secreted SPINK1 protein into culture medium regardless of oxygen conditions (Figure 4B) compared with its negative control cells with empty vector (DU145/EV). Most importantly, the DU145/SPINK1 stable transfectants exhibited radioresistance compared with DU145/EV cells in vitro (Figure 4C). The D_50_ value was significantly increased by the forced expression of SPINK1 in the stable transfectants from 3.49 ± 0.33 to 4.53 ± 0.69 Gy (Table 1). We then performed tumor growth delay assays using subcutaneous xenografted tumors grown from the stable transfectants and analyzed the effect of SPINK1 on tumor radioresistance in vivo. Although plasma SPINK1 levels were high in the DU145/SPINK1 tumor-bearing mice, as expected (Figure 4D), the secreted SPINK1 had no effect on the growth of the xenografted tumors without radiation treatment (Figure 4E and Table 2). Meanwhile, when the tumor xenografts were locally irradiated with γ-rays at a dose of 10 Gy, the SPINK1 overexpression enhanced tumor growth after the radiation treatment (Figure 4E). The time required for a 3-fold increase in tumor volume was significantly shorter in the case of DU145/SPINK1 (20.8 ± 3.7 days) than DU145/EV (27.8 ± 4.1 days) xenografts (Figure 4E and Table 2), suggesting that DU145/SPINK1 xenografts were more radioresistant than DU145/EV xenografts. All of these in vitro and in vivo data clearly suggest that SPINK1 protein secreted from hypoxic tumor cells induced tumor radioresistance in a paracrine manner and accelerates tumor growth after radiation therapy.

### SPINK1 decreased radiation-induced DNA damage and enhanced radioresistance of cancer cells in EGFR-dependent and nuclear factor erythroid 2–related factor 2–dependent (Nrf2-dependent) manners.

**Figure 5:**
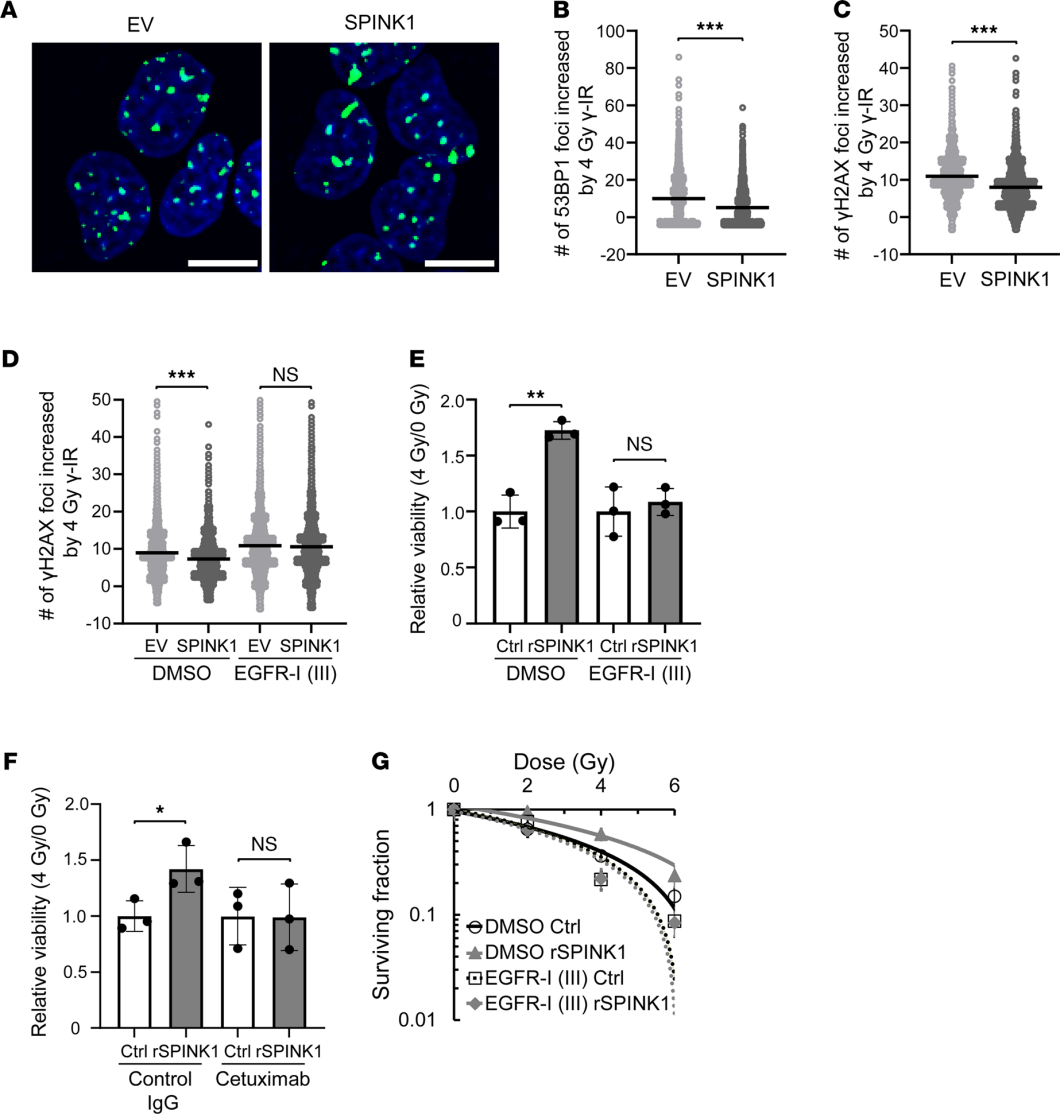
SPINK1 decreases radiation-induced DNA damage and enhances radioresistance of cancer cells in a EGFR-dependent manner. (**A**–**D**) Four days after being transfected with either pcDNA4/SPINK1 or its EV, DU145/EGFP-53BP1-M (**A** and **B**) and DU145 (**C** and **D**) cells were irradiated with 0 or 4 Gy of γ-rays in the presence or absence of EGFR-I III (**D**), and the DNA DSBs detected as EGFP-53BP1 foci (**A** and **B**) or as γH2AX foci (**C** and **D**) were analyzed 2 hours (**A** and **B**) or 15 minutes (**C** and **D**) after the radiation. (**A**) Immunocytochemical analysis. Green, EGFP-53BP1 foci; blue, counter staining using Hoechst 33342. Scale bar: 10 μm. (**B**–**D**) The number of foci increased by 4 Gy γ-IR was calculated by subtracting the number of foci at 0 Gy from that at 4 Gy under each condition and represented as dot plots with mean ± SD. (**E** and **F**) DU145 cells were irradiated with 0 or 4 Gy of γ-ray in the presence or absence of 100 ng/mL rSPINK1 in combination with DMSO or 0.5 μM EGFR-I III (**E**), or with control IgG or 10 μg/mL cetuximab (**F**), and subjected to colorimetric cell viability assays. (**G**) The same experiment as in Figure 3B was conducted in the presence or absence of EGFR-I III. Data are represented as mean (*n* > 1000 in **B**–**D**) and mean ± SD (*n* = 3 in **E** and **F**, and *n* = 6 in **G**). Two-tailed Student’s *t* test. *P < 0.05, **P < 0.01, ***P < 0.001. SPINK1, serine peptidase inhibitor Kazal type 1; EV, empty vector. EV, empty vector; EGFR-I III, EGFR Inhibitor III; DSBs, double-strand breaks.

Next, we addressed the molecular mechanism by which SPINK1 protected cancer cells from apoptotic cell death and enhanced tumor radioresistance. Because ionizing radiation induces apoptosis by producing various types of DNA damage including DNA double-strand breaks (DSBs), we first analyzed the potential of SPINK1 to protect genomic DNA from DSBs (Figure 5, A and B). The reporter gene, which expresses a fusion protein of EGFP and 53 binding protein 1 (EGFP-53BP1), and thus enables us to monitor DNA-DSBs as foci of green fluorescence, revealed that forced expression of SPINK1 partially but significantly reduced the number of DNA-DSBs caused by ionizing radiation (Figure 5, A and B). The same trend in results was confirmed in the so-called γH2AX focus assay based on an immunocytochemical analysis against another DNA-DSB marker, γH2AX (Figure 5C).

SPINK1 shows approximately 50% amino acid sequence similarity with EGF (24) and has been reported to bind to the EGFR and activate its downstream signaling cascade for the survival and proliferation of cells (25, 26). Thus, we tested the possibility that SPINK1 induced the radioresistance of cells in paracrine and EGFR-dependent manners. In the γH2AX focus assay using an EGFR inhibitor, EGFR Inhibitor III, SPINK1 did not exhibit a radioprotective effect when EGFR activity was inhibited (Figure 5D). Supportively, the EGFR-dependency of SPINK1 was also observed using another approach: rSPINK1 protein increased the viability of cells after 4 Gy γ-radiation, but not when EGFR activity was inhibited by EGFR Inhibitor III (Figure 5E) or by a chimeric monoclonal antibody against EGFR, cetuximab (Figure 5F). The clonogenic cell survival assay consistently demonstrated that rSPINK1 protein induced cancer cell radioresistance in the absence of EGFR Inhibitor III, but not in its presence (Figure 5G and Table 1).

**Figure 6:**
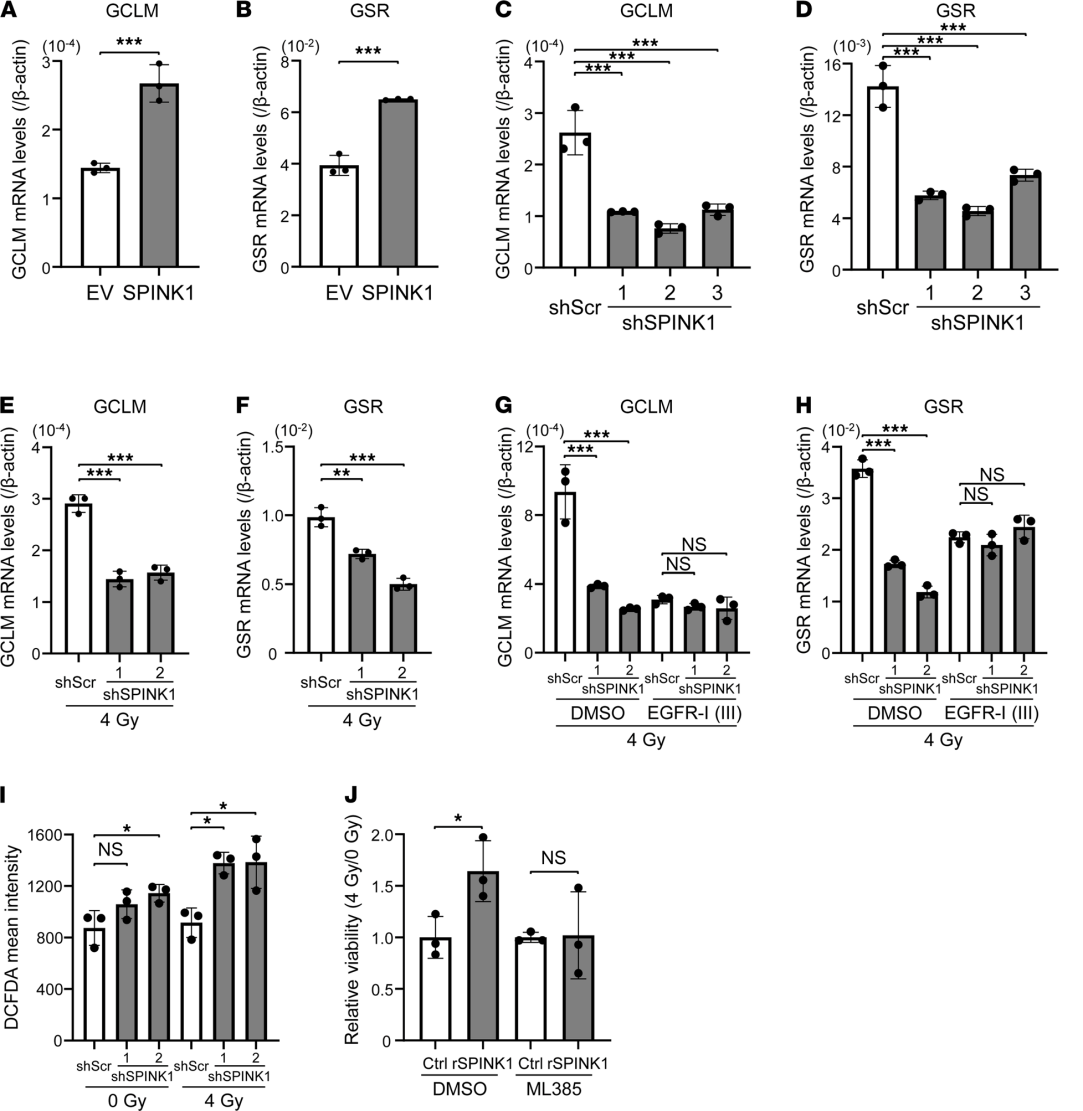
SPINK1 decreases radiation-induced DNA damage and enhances radioresistance of cancer cells in a NRF2-dependent manner. (**A** and **B**) Four days after being transfected with either pcDNA4/SPINK1 (SPINK1) or its EV, DU145 cells were subjected to qPCR. (**C**–**I**) HeLa/scramble cells and HeLa/shSPINK1-1, HeLa/shSPINK1-2, and HeLa/shSPINK1-3 cells were cultured under severe hypoxic conditions (O_2_ < 0.1%) for 48 hours, irradiated with 0 (**C**, **D**, and **I**) or 4 (**E**–**I**) Gy of γ-rays and subjected to qPCR (**C**–**H**) or the DCFDA cellular ROS assay (**I**). Cells were irradiated in the presence or absence of the EGFR-I III (**G** and **H**). (**J**) DU145 cells were irradiated with γ-rays in the presence or absence of 100 ng/mL rSPINK1 in combination with DMSO or 2 μM ML385 and subjected to the colorimetric cell viability assay. Data are represented as mean ± SD (*n* = 3 in **A**–**J**). Two-tailed Student’s *t* test (**A**, **B**, and **J**). One-way ANOVA with Dunnett’s test (**C**–**I**). *P < 0.05, **P < 0.01, ***P < 0.001. SPINK1, serine peptidase inhibitor Kazal type 1; EV, empty vector; DCFDA, dichlorodihydrofluorescein diacetate; EGFR-I III, EGFR Inhibitor III.

Next, we aimed to identify the relevant downstream factors of EGFR. We focused on a transcription factor for the expression of a series of antioxidant genes, Nrf2, because it has been reported to be activated downstream of EGFR-mediated signaling (27). Ectopic expression of SPINK1 significantly enhanced the mRNA levels of Nrf2 target antioxidant genes, including glutamate-cysteine ligase modifier subunit (GCLM) and glutathione reductase (GSR), as expected (Figure 6, A and B). Consistently, loss-of-function studies demonstrated that silencing of the SPINK1 gene significantly suppressed the mRNA levels of both GCLM and GSR in nonirradiated (0 Gy) and irradiated (4 Gy) cells under hypoxic conditions (Figure 6, C–F, Supplemental Figure 8, and Supplemental Figure 9, A and B). The suppressing effect was not observed in the presence of EGFR Inhibitor III, indicating that expressions of the Nrf2 target antioxidant genes are induced downstream of SPINK1-EGFR–mediated signaling (Figure 6, G and H, and Supplemental Figure 9, C and D). To examine whether SPINK1 induces antioxidant properties, we employed a cell-permeable fluorescent probe for ROS, dichlorodihydrofluorescein diacetate (DCFDA). The DCFDA assay showed that knockdown of SPINK1 resulted in a significant increase of intracellular ROS levels after 4 Gy irradiation (Figure 6I). Finally, we examined the involvement of the Nrf2-related expression of antioxidant genes in SPINK1-dependent cancer radioresistance. A colorimetric cell viability assay clearly demonstrated that the radioresistance caused by rSPINK1 was almost completely abrogated with an Nrf2 inhibitor, ML385 (Figure 6J). All of these data suggest that SPINK1 decreased radiation-induced DNA damage by upregulating EGFR-mediated and Nrf2-dependent antioxidant responses, and consequently induced cancer radioresistance.

### SPINK1 was a predictive plasma marker for tumor hypoxia.

Our in vitro data showed that the amount of secreted SPINK1 protein increased according to the induction of its mRNA expression upon hypoxia, which led us to hypothesize that SPINK1 protein could be a good plasma marker to monitor the hypoxic burden in a malignant solid tumor. To test this possibility, we conducted the following 4 kinds of studies.

**Figure 7:**
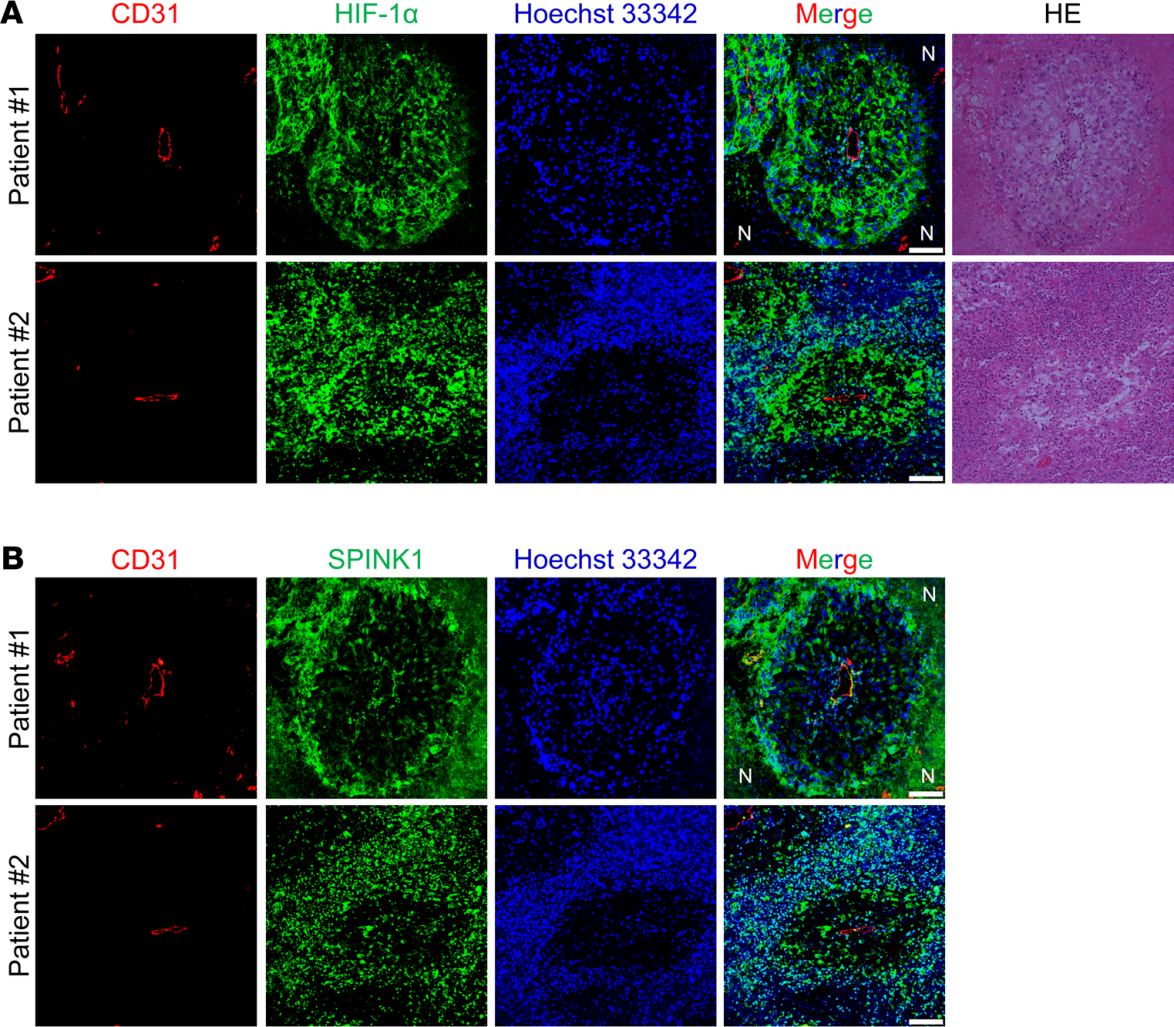
SPINK1 protein is expressed in severely hypoxic regions and secreted to the proximal regions of blood vessels. (**A** and **B**) Two pairs of serial sections of clinical human ccRCC tissues from 2 independent patients were stained with the indicated antibodies in **A** and **B**, respectively. Corresponding serial sections were stained with H&E. Scale bar: 50 μm. N, necrosis. SPINK1, serine peptidase inhibitor Kazal type 1; EV, empty vector; ccRCC, clear cell renal cell carcinoma.

First, we examined whether SPINK1 protein is expressed in hypoxic regions and secreted to the surrounding and oxygenated regions in human cancers by performing IHC analysis. We intentionally used clear cell renal cell carcinoma tissues, approximately 95% of which harbor deficiency in functional VHL, to analyze whether SPINK1 expression depends on the VHL–HIF-1 axis in human cancers. As expected, HIF-1α protein was detected in the entire tumor tissue regardless of the distance from tumor vessels, except for necrotic regions (Figure 7A). SPINK1 proteins were also observed in the tumor tissue, but it is noteworthy that SPINK1 protein did not exhibit a homogeneous expression pattern; a strong signal was predominantly detected in perinecrotic regions distal to blood vessels (Figure 7B). In addition to these in vivo data, our in vitro data showing that HIF-dependent SPINK1 expression was induced upon severe hypoxia, but not upon mild hypoxia, collectively suggest the presence of a mechanism by which the function of HIFs is suppressed specifically in the SPINK1 gene locus under milder hypoxic conditions. Although the mechanism remains unknown, these results suggest that SPINK1 protein was expressed in severely hypoxic regions and secreted to the proximal regions of blood vessels. Importantly, SPINK1 protein could be detected around the CD31-positive endothelial cells (Figure 7B).

**Figure 8:**
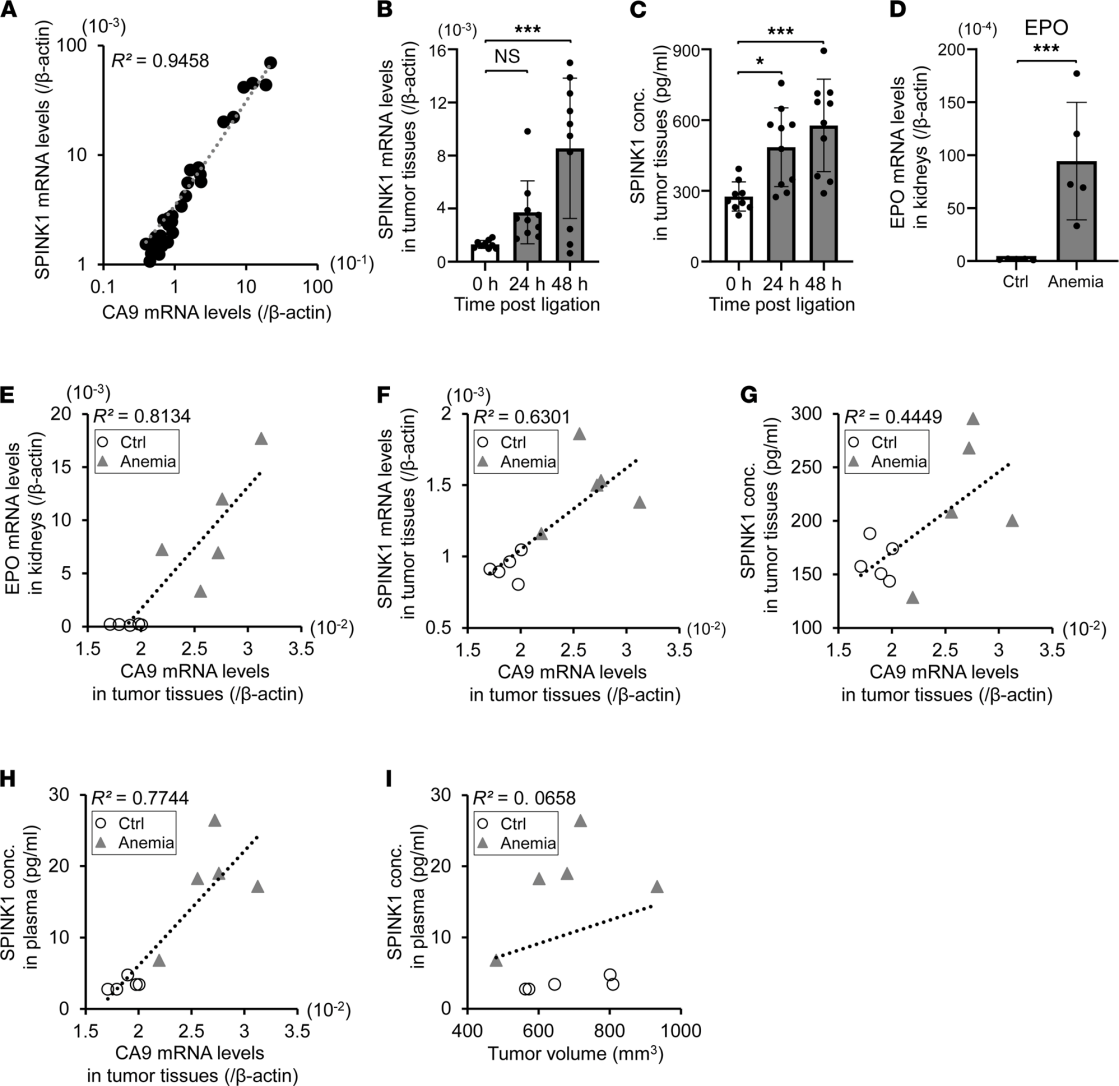
SPINK1 in plasma reflects the degree of hypoxia within tumor tissue in vivo. (**A**) A scatter plot for correlation analysis between mRNA levels of SPINK1 and CA9 in 36 HeLa tumor xenografts showed a good coefficient of determination, *R*^2^ = 0.9458. (**B** and **C**) After blood flow to the HeLa tumor xenografts was decreased by ligaturing the leg for the indicated times, levels of SPINK1 mRNA (**B**) and SPINK1 protein (**C**) in the tumor tissues were quantified by qPCR and the ELISA assay, respectively. (**D**–**I**) After anemia treatment by phenylhydrazine administration, mRNA levels of EPO in the kidneys (**D** and **E**) and those of CA9 (**E**–**H**) and SPINK1 (**F**) in tumor tissues were quantified by qPCR. SPINK1 protein levels in tumors (**G**) and plasma (**H** and **I**) and the tumor volume (**I**) were measured by the ELISA assay and digital calipers, respectively. Scatter plots for correlation analysis between the 2 indicated factors (**E**–**I**). Data are represented as mean ± SD (**B**–**D**; *n* = 36 in **A**, *n* = 9–10 in **B** and **C**, *n* = 5 in **D**–**I**). Two-tailed Student’s *t* test (**D**). One-way ANOVA with Dunnett’s test (**B** and **C**). *P < 0.05, ***P < 0.001. SPINK1, serine peptidase inhibitor Kazal type 1; EV, empty vector; CA9, carbonic anhydrase 9; EPO, erythropoietin.

Next, we compared the mRNA levels of SPINK1 with those of one of the most representative intrinsic hypoxia markers, CA9, in 36 subcutaneous HeLa tumor xenografts by qPCR. The resulting scatter plot showed a good correlation between their expressions, with *R*^2^ = 0.9458 (Figure 8A).

Next, we examined whether SPINK1 was induced when blood supply to a xenograft was reduced by occluding vasculature to the tissue. Consistent with the in vitro data presented, qPCR and the ELISA assay showed that the levels of SPINK1 mRNA and SPINK1 protein in the xenografted tumors were significantly increased according to the duration of ligation (Figure 8, B and C). However, we could not detect SPINK1 protein circulating in plasma after the ligation, probably because the occlusion prevented SPINK1 proteins from being systemically released from hypoxic tumor cells (data not shown).

As another in vivo experimental model, by which oxygen supply, but not blood supply, to a xenografted tumor was reduced, we next employed an anemia mouse model using a hemolytic reagent, phenylhydrazine (PHZ). PHZ injection significantly facilitated the mRNA expression of both erythropoietin in kidneys and the intrinsic hypoxia marker, CA9, in tumors (Figure 8, D and E). Their expression levels exhibited a positive correlation with each other (Figure 8E; *R^2^* = 0.8134), suggesting that the oxygen supply to peripheral tissues including xenografted tumors was reduced in this acute anemia mouse model. In this experimental setting, anemia treatment induced more expressions of SPINK1 mRNA and SPINK1 protein in tumor tissues according to the extent of tumor hypoxia, monitored as the CA9 mRNA levels (Figure 8, F and G; *R^2^* = 0.6301 and *R^2^* = 0.4449, respectively). Moreover, plasma SPINK1 levels were positively correlated with the extent of tumor hypoxia (Figure 8H; *R^2^* = 0.7744), but not with the volume of xenografted tumors (Figure 8I; *R^2^* = 0.0658). Of note, when we applied the anemia model to nontumor-bearing mice, plasma SPINK1 levels were not increased at all (Supplemental Figure 10), suggesting that circulating SPINK1 protein originated from the tumor xenografts, but not from other normal tissues. All of these data demonstrate that SPINK1 in plasma reflected the degree of hypoxia within tumor tissue in vivo and that SPINK1 could have been a good plasma marker for tumor hypoxia.

## Discussion

In the present study, we successfully identified SPINK1 as a hypoxia-responsive secretory protein and revealed that its expression is induced at the transcriptional level in a HIF-dependent manner. Moreover, we demonstrated that SPINK1 secreted from hypoxic cancer cells has the potential to induce radioresistance of the surrounding oxygenated cells in a paracrine manner through activation of the EGFR-mediated and Nrf2-mediated antioxidant pathway. These data combined with our in vivo data showed that SPINK1 levels detected in plasma were correlated with the hypoxic burden in a xenograft model. All the data indicate that SPINK1 can be utilized as a predictive plasma marker for the tumor hypoxic fraction and therapeutic effect of radiation. Furthermore, because an anti-SPINK1 neutralizing antibody revealed a radioprotective effect of SPINK1, the present study indicates the usefulness of SPINK1 as a therapeutic target for radiosensitization as well.

We found that SPINK1 expression was induced at the transcription initiation level by not only hypoxic stimuli but also treatment with PHD inhibitors or a proteasome inhibitor. These results collectively indicate that HIFs, whose activities are negatively regulated by PHDs and the proteasome pathway in the presence of oxygen, are factors responsible for hypoxia-dependent SPINK1 expression. Loss-of-function studies of HIFs further strengthened this conclusion. However, our in vitro studies using the qPCR technique revealed that mRNA expression of SPINK1 was markedly induced upon severe hypoxia (0.1% O_2_) but not upon milder hypoxia (1%–10% O_2_), although that of a representative HIF-1 downstream factor, CA9, began to be induced even upon mild hypoxia. Moreover, IHC staining using clinical RCC tissues demonstrated that, whereas HIF-1α was expressed in entire tumor tissue including the proximal regions of tumor vessels due to the loss of VHL, SPINK1 expression was predominantly detected in perinecrotic regions distal to blood vessels. These in vitro and clinical data suggest the presence of an unknown mechanism, by which the function of HIFs is suppressed specifically in the SPINK1 gene locus only under milder hypoxic conditions.

When blood flow to the xenografted tumors was decreased by ligaturing the tumor-bearing leg or by anemia treatment with PHZ injection, the expressions of SPINK1 mRNA and SPINK1 protein were significantly induced in our in vivo studies. In addition to these data, our in vitro data showing that SPINK1 expressions were induced upon severe hypoxic treatment in HIF-dependent manner supported our suggestion that SPINK1 can be utilized as a hypoxia marker. However, it should be noted that there still remains a possibility that inflammatory stimulation, etc., caused by ligaturing or anemia treatment, but not hypoxic stimuli, might have induced the SPINK1 expression.

Tumor hypoxia, particularly severe hypoxia and anoxia, has been strongly associated with malignant phenotypes and therapy resistance of cancer cells and the poor prognosis of cancer patients. Therefore, considerable efforts have been devoted to the development of a strategy quantifying the hypoxic tumor fraction and for its application to personalized cancer therapy. Because the present study revealed that SPINK1 was secreted from cancer cells under severe hypoxic conditions to plasma, we can expect to be able to utilize SPINK1 as a convenient plasma hypoxia marker. To realize this, it is critical to examine whether plasma SPINK1 levels are positively correlated with the tumor hypoxic fraction in not only tumor-bearing mice, as confirmed here, but also cancer patients.

Whether SPINK1 protein levels are above detectable levels in plasma of cancer patients is another important issue to exploit it as a biomarker of the hypoxic tumor burden. Chen et al. previously reported that SPINK1 protein is detectable in peripheral blood of cancer patients (28). In the present study, our focus is solely on providing the first in vitro and in vivo evidence supporting the utility of SPINK1 as a plasma biomarker for tumor hypoxia, and we will report the results of analysis with cancer patients in our next paper.

Accumulating evidence has shown that SPINK1 is associated with tumor malignant phenotypes, such as proliferation, angiogenesis, migration, invasion, metastases, and antiapoptosis (22, 23, 29–35). In the present study, we identified its function in tumor radioresistance. SPINK1 overexpression led to a radioprotective effect in cells under normoxic and mildly hypoxic (but not under severely hypoxic) conditions in the present in vitro clonogenic survival assay. Treatment with the recombinant SPINK1 protein and the forced expression of SPINK1, but not that of SPINK1-ΔSP, significantly increased the viability of cancer cells after radiation in vitro, indicating that SPINK1 induced radioresistance in a paracrine manner. In addition, because SPINK1 proteins were found to diffuse from hypoxic areas toward relatively oxygenated layers, our data collectively indicate that SPINK1 proteins secreted by cancer cells in hypoxic layers protected neighboring cancer cells in the relatively oxygenated layers from radiation. Based on these findings, we propose the use of a neutralizing antibody against SPINK1 to overcome the radioresistance of cancers.

Our colorimetric cell viability assay demonstrated that the radioresistance caused by SPINK1 was almost completely abrogated by the inhibition of EGFR. Interestingly, Nrf2 inhibition reduced the radioresistance to the same extent as EGFR inhibition, suggesting that EGFR and Nrf2 functioned in the same pathway upon stimulation by SPINK1. These results are consistent with previous reports that SPINK1 binds to EGFR and subsequently activates its downstream signaling cascade and that Nrf2 is one of the EGFR downstream genes (25–27).

It has been repeatedly reported that SPINK1 is overexpressed in various types of cancer tissues, such as gastrointestinal, genitourinary, gynecologic, liver, lung, and breast cancers (35). This observation may be reasonable from the viewpoint of hypoxia biology as hypoxic regions develop in most malignant solid tumors and SPINK1 expression is induced upon hypoxia, as revealed here. However, previous studies reported an interesting phenomenon, whereby some cell lines, such as a prostate cancer–derived cell line, 22RV1, and a colon cancer–derived cell line, WiDr, highly express SPINK1 even under normoxic conditions (32, 36, 37). In such a case, unknown mechanisms might cause the aberrant expression. If this is the case, we may not be able to call SPINK1 a hypoxia marker in this kind of case but can still utilize it at least as a predictive marker of the therapeutic effect of radiation due to its radioprotective effect.

SPINK1 was originally identified as a trypsin inhibitor serving to cleave prematurely activated trypsin protein and prevent the enzyme from causing damage to the pancreas. Therefore, systematic inhibition of SPINK1 might increase the risk of potential adverse side effects, particularly pancreatitis (35, 38). To avoid such a problem, further studies are needed, e.g., developing a drug delivery system to carry a SPINK1 inhibitor specifically toward malignant tumor tissues, and elucidating the difference in molecular mechanism behind the expression of SPINK1 between normal pancreas tissue and malignant tumor tissue. By further addressing these issues, it is expected that the present research can open a new avenue toward the development of a strategy for personalized cancer therapy using SPINK1.

## Methods

### Cell culture.

A human cervical epithelial adenocarcinoma cell line, HeLa, a human prostate adenocarcinoma cell line, DU145, and a human osteosarcoma cell line, U2OS, were purchased from the American Type Culture Collection. An immortalized human embryonic kidney cell line, HEK293TN, was purchased from System Biosciences. Cells were cultured at 37°C in DMEM containing 10% FBS, 100 U/mL penicillin, and 100 μg/mL streptomycin. Cells were incubated in well-humidified 5% CO_2_ and 95% air for the normoxic culture, in RUSKINN INVIVO2 400 (Ruskinn Technology Limited) for mild hypoxic culture at 1%–10% O_2_, or in RUSKINN INVIVO2 500 (Ruskinn Technology Limited) for severe hypoxic culture at less than 0.1% O_2_.

### Reagents.

Plasmids and siRNA were transiently transfected with Polyfect Transfection Reagent (QIAGEN) and Lipofectamine RNAiMAX Transfection Reagent (Thermo Fisher Scientific), respectively, according to the manufacturers’ instructions. All siRNAs were purchased from Thermo Fisher Scientific and their target sequences are listed in Supplemental Table 1. Recombinant SPINK1 (rSPINK1; Abnova), EGFR inhibitors, EGFR Inhibitor III (MilliporeSigma), cetuximab (Carbosynth), an Nrf2 inhibitor, ML385 (MilliporeSigma), anti-SPINK1 mouse monoclonal antibody (MoBiTec, clone R XXIII, catalog PSKAN2), deferoxamine (MilliporeSigma), DMOG (MilliporeSigma), MG132 (MilliporeSigma), and Act D (Nacalai Tesque) were used in the present study.

### Plasmid construction.

To construct pcDNA4/SPINK1, a DNA fragment encoding the human *spink1* gene was amplified from cDNA of HeLa cells by PCR using the SPINK1 forward primer (5′-ATAGGATCCGCCATGAAGGTAACAGGCATC-3′) and the SPINK1 reverse primer (5′-GGCGAATTCGCAAGGCCCAGATTTTTGAAT-3′), and inserted between the BamHI and EcoRI sites of pcDNA4/myc-His A (MilliporeSigma). pcDNA4/SPINK1-Δ signal peptide (herein pcDNA4/SPINK1-ΔSP) was basically constructed through the same procedure as above but particularly using the following forward primer 5′-TATGGATCCGCCATGGACTCCCTGGGAAGAG-3′. To construct pCDH/SPINK1, a DNA fragment encoding the human *spink1* gene was amplified from pcDNA4/SPINK1 using the following primers: 5′- ATAGAATTCGCCGCCATGAAGGTAACAGGC-3′ and 5′-ATGCGGCCGCTCAGCAAGGCCCAGATTTTTG-3′, and inserted between the EcoRI and NotI sites of pCDH-EF1-MCS-IRES-Puro (System Biosciences). To construct pCDH/EGFP-53BP1M, a DNA fragment encoding EGFP-53BP1M was prepared from pEGFP-53BP1M (21) by digesting it with NheI and BamHI, and inserted between the corresponding sites of pCDH-EF1-MCS-IRES-Puro. Plasmids expressing shRNA against SPINK1 were prepared using pRSI12-U6-sh-HTS4-UbiC-TagRFP-2A-Puro (Cellecta) based on information about shRNA against SPINK1 in the Cellecta DECIPHER shRNA Library (catalog DHDAC-M2-P). The same vector expressing scrambled nontargeting control shRNA was purchased from Cellecta. The targeting sequences are listed in Supplemental Table 2.

### Stable transfectants.

HEK293TN cells were transfected with pCDH/EGFP-53BP1M; pCDH/SPINK1 or pCDH/EV; pRSI12/shSPINK1-1, pRSI12/shSPINK1-2, or pRSI12/shSPINK1-3; or pRSI12/shScr for the production of lentiviruses encoding the EGFP-53BP1M reporter cassette, expression cassette for SPINK1 or none, shRNA against SPINK1-1, SPINK1-2, or SPINK1-3, or scramble RNA. Then, DU145 and HeLa cells were infected with each virus and cultured with puromycin to establish DU145/EGFP-53BP1M, DU145/SPINK1, DU145/EV, HeLa/shSPINK1-1, HeLa/shSPINK1-2, HeLa/shSPINK1-3, and HeLa/scramble cells, accordingly.

### Microarray gene expression analysis.

Microarray gene expression analysis was performed using a GeneChip system with a Human Genome U133-plus 2.0 array, which was spotted with 54,675 probe sets (Affymetrix Inc.), according to the manufacturer’s instructions. In brief, 500 ng of total RNA was used to synthesize cRNA with a GeneChip 3′ IVT Express Kit (Affymetrix Inc.). Fragmented biotin-labeled cRNA was hybridized to the array at 45°C for 16 hours. After the staining with streptavidin-phycoerythrin, the array was scanned using a probe array scanner. The obtained hybridization intensity data were analyzed using GeneSpring GX software (Agilent Technologies Inc.) to extract the significant genes. The microarray data set was deposited in the NCBI’s Gene Expression Omnibus database (GEO GSE161393).

### Irradiation.

Cultured cells and xenografted tumors in the right hind legs of nude mice were irradiated with the indicated dose of ^137^Cs γ-rays using Gammacell 40 Exactor (MDS Nordion International Inc.).

### Western blotting.

The indicated cells were transfected with the indicated plasmids and cultured for 2 days in 0.1% FBS-containing medium under normoxic conditions. Both cell lysates harvested with CelLytic M (MilliporeSigma) and culture medium were subjected to Western blotting using anti-myc epitope tag mouse monoclonal antibody (1000-fold dilution; Cell Signaling Technology, clone 9B11, catalog 2276) for the detection of exogenously expressed SPINK1 and its derivatives and anti-human β-actin mouse monoclonal antibody (200-fold dilution; Santa Cruz, clone AC-15, catalog Sc-69879) as primary antibodies, anti-mouse IgG HRP-linked whole Ab (5000-fold dilution; GE Healthcare Bioscience) as secondary antibody, and ECL Prime Western Blotting Detection Reagents (GE Healthcare Bioscience) for detection. The culture media were 17 times concentrated using Amicon Ultra-0.5 mL Centrifugal Filters, Ultracel-3K (Merck Millipore) before Western blotting according to the manufacturer’s instructions.

### ELISA assay in vitro.

After the indicated cells were cultured in 1.5 mL DMEM under normoxic or severe hypoxic conditions (O_2_ = 20% or < 0.1%, respectively) for 2 days (1.5–2.0 × 10^5^ cells/well in a 6-well plate), the cell lysate harvested with 100 μL CelLytic M and the culture medium were subjected to NanoDrop (Thermo Fisher Scientific) to quantify the concentration of total protein and the ELISA assay to quantify the secreted SPINK1, respectively. The concentration of SPINK1 in the culture medium was normalized with the concentration of the total protein in the cells. The ELISA assay was conducted using Human SPINK1 DuoSet ELISA according to the manufacturer’s instructions (R&D Systems).

### Colorimetric cell viability assay.

After 24 hours serum starvation, DU145 cells (400 cells/well for 0 Gy radiation and 1200 cells/well for 4 Gy radiation in 96-well plates) were precultured under normoxic conditions in the presence or absence of 100 ng/mL rSPINK1 in combination with or without 0.5 μM EGFR Inhibitor III, 10 μg/mL Cetuximab, or 2 μM ML385 for 24 hours. To neutralize SPINK1, the rSPINK1-containing medium was preincubated at 37°C for 1 hour with anti-SPINK1 monoclonal mouse antibody (MoBiTec, clone R XXIII, catalog PSKAN2) or control IgG (Mouse C57BL6 IgG Affinity Purified, Innovative Research, catalog IMSC57IGGAP10MG). Then, the cells were treated with 0 or 4 Gy γ-ray irradiation, cultured under normoxic conditions for 3 additional days, and subjected to the cell count assay using Cell Count Reagent SF (Nacalai Tesque) to quantify cell viability after irradiation. The viability of cells after 4 Gy radiation was divided by that after 0 Gy to calculate the viability of cells after radiation. The value obtained in the presence of rSPINK1 treatment (rSPINK1 group) was further divided by that obtained in its absence (control group) to calculate the relative viability (4 Gy/0 Gy), shown in Figure 3, C and L; Figure 5, E and F; and Figure 6J.

### FACS analysis.

After 24 hours serum starvation, DU145 cells (5.0 × 10^4^ cells/well in a 6-well plate) were precultured under normoxic conditions (O_2_ = 20%) in the presence or absence of 100 ng/ml rSPINK1 for 24 hours, and irradiated with 0 or 4 Gy γ-ray. Three days later, the cells were subjected to flow cytometry using BD FACS CantoII (BD Bioscience) to analyze the cell cycle status and quantify the percentage of cells in the sub-G_1_ fraction, as previously described (21, 39).

### Quantitative real-time PCR.

After the indicated cells (1.0 × 10^5^ cells/well in a 6-well plate) were cultured under normoxic, mild hypoxic, or severe hypoxic conditions for the indicated periods, total RNA was extracted and subjected to reverse transcription followed by quantitative real-time PCR to quantify mRNA levels of the indicated genes, as previously described (40). All primers are listed in Supplemental Table 3. Human or mouse ACTB mRNA levels were used as an internal control.

### Clonogenic survival assay.

After the indicated pretreatment, cells were irradiated with the indicated doses of γ-rays, and cultured for 2 additional weeks. Surviving colonies were fixed with 70% ethanol and stained with Giemsa solution. Surviving fractions were calculated, as previously described (41, 42).

### DCFDA cellular ROS assay.

The assay was performed according to the manufacturer’s instructions, as previously described (43).

### In vivo experiments.

Tumor-bearing mice were prepared by transplanting suspensions of the indicated cancer cells into right hind legs of 8–10-week-old female nude mice (BALB/c nu/nu; SLC Inc.). To increase hypoxic fractions in HeLa tumor xenografts, oxygen supply to the xenografts was reduced by directly ligating the tumor-bearing leg for the indicated periods, or by i.p. injecting a hemolytic reagent, phenylhydrazine hydrochloride (60 mg/kg of body weight, MilliporeSigma), twice with a 1-day interval, and the tumor tissues and peripheral blood samples were harvested. After the entire tumor tissues were minced with Tissue Lyser LT (QIAGEN), total RNA and total protein were extracted using Sepasol RNA I Super G (Nacalai Tescue) and CelLytic M for qPCR and the ELISA assay, respectively. Peripheral blood samples were centrifuged in EDTA tubes at 1200*g* for 10 minutes at 4°C to quantify SPINK1 protein levels in plasma by the ELISA assay using Human SPINK1 DuoSet ELISA (R&D Systems). For the growth delay assay, the indicated xenografts (tumor volume, ~150–200 mm^3^) were locally irradiated with 10 Gy ^137^Cs γ-rays. Tumor volumes were calculated as 0.5 × length × width^2^ and compared with the initial value to calculate the relative tumor volume.

### IHC analyses.

Tumor xenografts were surgically excised 60 minutes after an i.p. injection of pimonidazole hydrochloride included in the Hypoxyprobe-1 Plus kit (Hypoxyprobe, Inc.). Formalin-fixed and paraffin-embedded sections of the tumor tissues were subjected to IHC staining using antipimonidazole mouse monoclonal antibody conjugated with FITC (Hypoxyprobe, Inc., clone 4.3.11.3, catalog HP2-100), anti-SPINK1/p12 rabbit monoclonal antibody (Abcam, clone EPR12696[2], catalog ab183034), anti-CD31 mouse monoclonal antibody (Abcam, clone: JC/70A, catalog ab9498), and anti–HIF-1α rabbit polyclonal antibody (Novus Biologicals, catalog NB100-479) as the first antibody, and using Alexa Fluor 488 or 594 goat anti-rabbit IgG and Alexa Fluor 594 donkey anti-mouse IgG (Thermo Fisher Scientific) as the second antibody (to detect SPINK1), as previously described (11, 21, 44). The reproducibility of each staining was confirmed at least 3 times in independent tumors, and representative results are shown.

### Immunocytochemical analyses.

The indicated cultured cells (6 × 10^3^ cells/well; in a 96-well black plate) were transiently transfected with pcDNA4/SPINK1 or its empty vector and precultured for 3 days with or without 0.5 μM EGFR Inhibitor III. The cells were irradiated with 0 or 4 Gy γ-rays and fixed with 4% paraformaldehyde 2 hours after (for DU145/EGFP-53BP1-M) or 15 minutes after (for DU145) the irradiation. DU145 cells were permeabilized with 0.2% Triton X-100 in PBS for 5 minutes, incubated in blocking solution (2% BSA in PBS) for 30 minutes, and treated with an anti-γH2AX [pSer139] rabbit polyclonal antibody (Novus Biologicals, catalog NB100-384) and with Alexa Fluor 488 goat anti-rabbit IgG (Thermo Fisher Scientific). The resultant foci were detected using IN Cell Analyzer 2000 (Cytiva) and analyzed using IN Cell Developer Toolbox.

### Statistics.

The significance of differences between 2 independent subjects and among multiple independent subjects was determined using 2-tailed Student’s *t* test and 1-way ANOVA with Dunnett’s test, respectively. A *P* value of less than 0.05 was considered significant. Representative data are presented from at least 3 independent experiments.

### Study approval.

All animal experiments were approved by the Animal Research Committee of Kyoto University and conducted according to the guidelines on animal experiments in Japan. The protocol of this study using human renal cancer samples was approved by the Ethics Committee of Kyoto University Hospital. Written-informed consent was obtained from each patient. The clinical study was carried out in accordance with the Helsinki Declaration. All samples were obtained from patients diagnosed with clear cell renal cell carcinoma at the Kyoto University Hospital.

## Author contributions

TS mainly performed the experiments, analyzed the data, and cowrote the manuscript. MK supervised the study and contributed to data analysis and critical discussion. YS contributed to a part of the mechanism study. YT performed the DNA microarray analysis. SA and OO contributed to the research using human renal cancer samples. JMN, NT, TM, and EMH contributed to critical discussion and cowrote the manuscript. HH designed and supervised the study, analyzed the data, and cowrote the manuscript.

## Supplementary Material

Supplemental data

## Figures and Tables

**Table 1:**
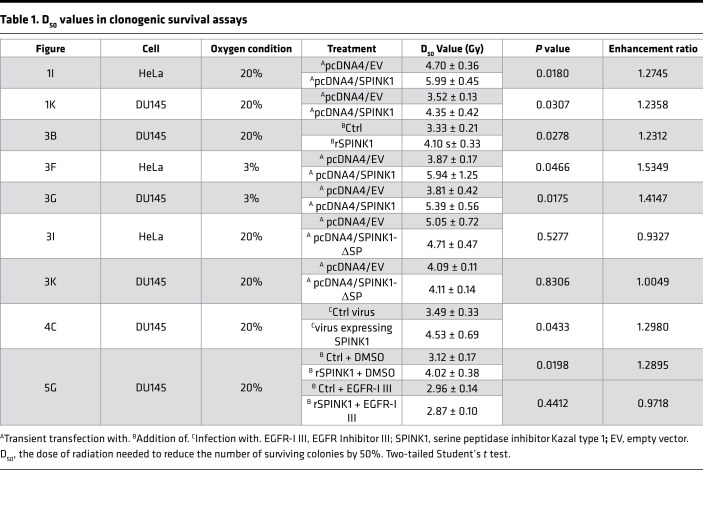
D_50_ values in clonogenic survival assays

**Table 2:**
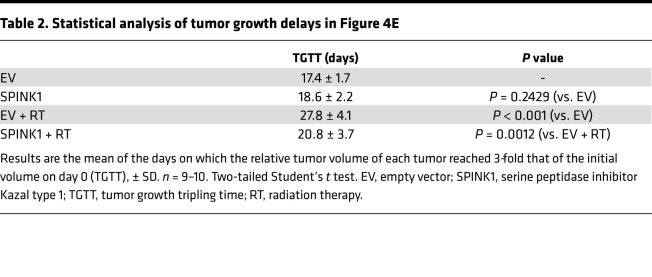
Statistical analysis of tumor growth delays in Figure 4E

## Version 1 11/08/2021

Electronic publication
