# Supplementary material for: SPINK1 as a plasma marker for tumor hypoxia and a therapeutic target for radiosensitization
Source: JCI Insight. 2021 Nov 8;6(21):e148135. doi: 10.1172/jci.insight.148135 (PMC8663551; doi:10.1172/jci.insight.148135)
Supplement: Supplemental data [file jciinsight-6-148135-s011.pdf]

Supplementary Information

SPINK1 as a plasma marker for tumor hypoxia and a therapeutic target for radiosensitization

Tatsuya SUWA, Minoru KOBAYASHI, Yukari SHIRAI, Jin-Min NAM, Yoshiaki TABUCHI, Norihiko TAKEDA, Shusuke AKAMATSU, Osamu OGAWA, Takashi MIZOWAKI, Ester M. HAMMOND, and Hiroshi HARADA

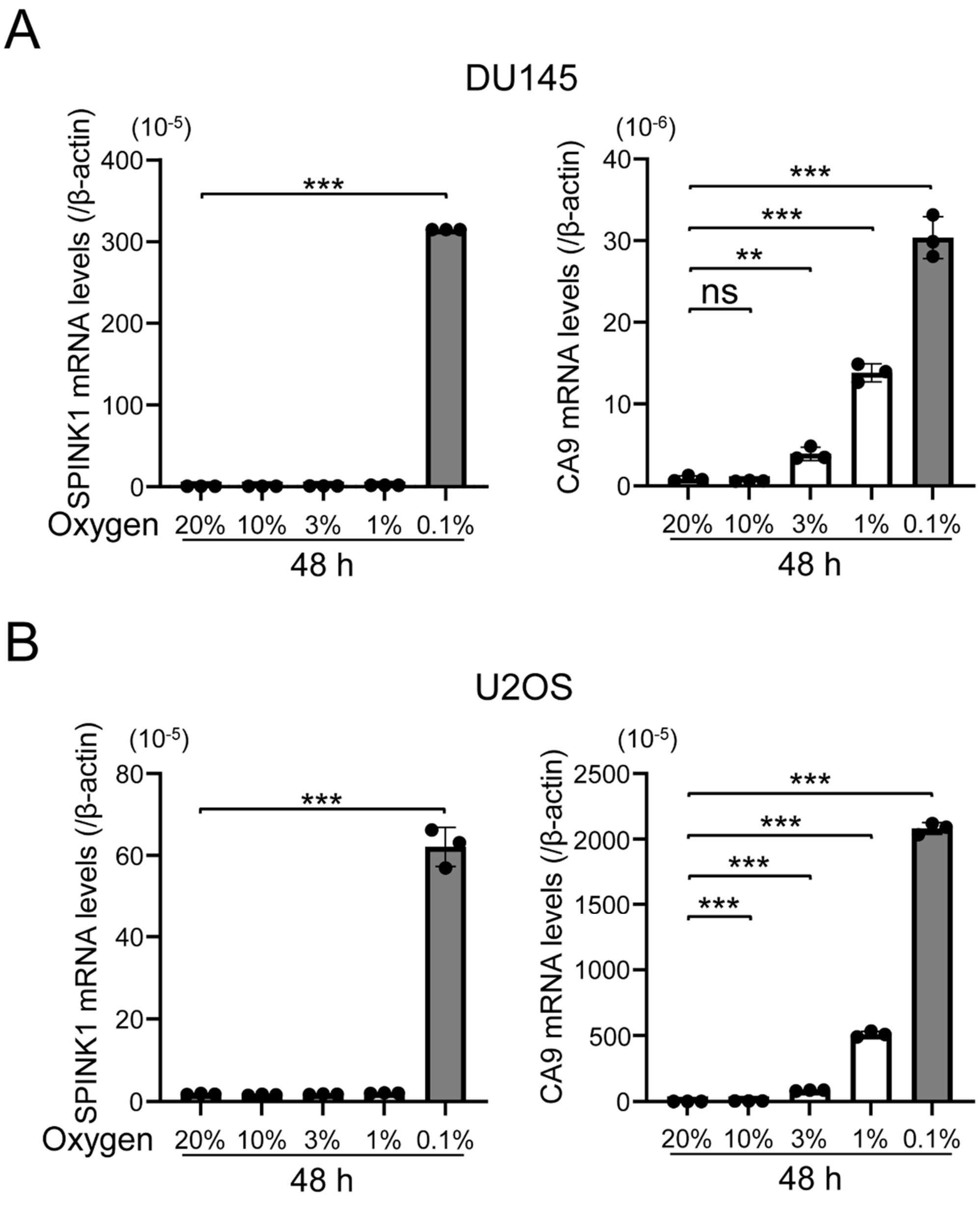

Supplemental Figure 1. Induction of SPINK1 mRNA expression under severely hypoxic conditions in various cancer cell lines. The indicated cells were cultured under the indicated oxygen conditions for 48 h, and subjected to qRT-PCR for the indicated genes. Data represent means ± SD (n = 3). ***P* < 0.01, ****P* < 0.001. ns, not significant.

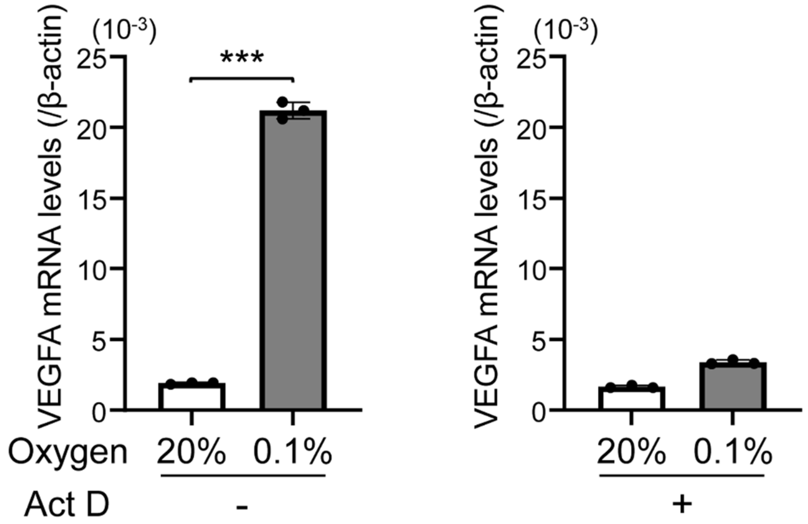

Supplemental Figure 2. The actinomycin D treatment suppressed transcription. HeLa cells were cultured under normoxic (O_2 = 20%) or severely hypoxic (O_2 < 0.1%) conditions for 24 h in the presence or absence of 5 μg/mL Act D, and VEGFA mRNA levels was quantified by qRT-PCR. Data represent means ± SD (n = 3). ****P* < 0.001.

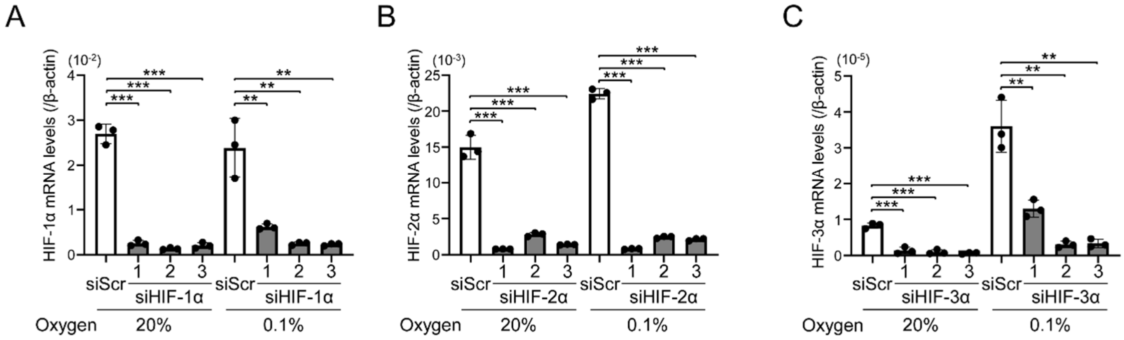

Supplemental Figure 3. Silencing of HIF-αs, HIF-1α, HIF-2α, and HIF-3α. (**A**-**C**) After silencing each HIF-1α (**A**), 2α (**B**), and 3α (**C**), HeLa cells were cultured under the indicated oxygen conditions for 24 h, and subjected to qRT-PCR to analyze the knockdown efficiency. Data represent means ± SD (n = 3). **P < 0.01, ***P < 0.001.

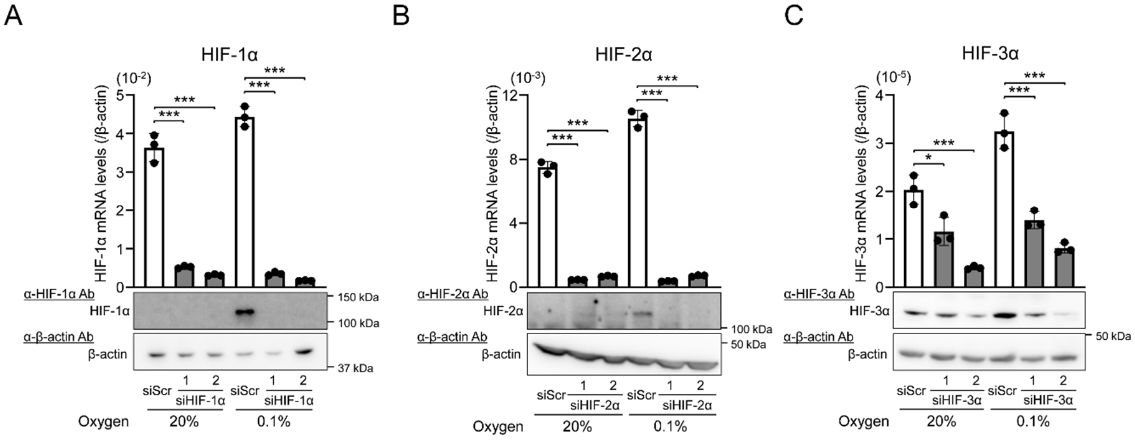

Supplemental Figure 4. Silencing of HIF-αs, HIF-1α, HIF-2α, and HIF-3α. (**A**-**C**) After silencing HIF-1α (**A**), 2α (**B**), and 3α (**C**), HeLa cells were cultured under the indicated oxygen conditions for 24 h, and subjected to qRT-PCR and Western blotting using the anti-human HIF-1α mouse monoclonal Ab (BD Transduction Laboratories, clone: 54, Cat. #610959), anti-human EPAS-1/HIF-2α mouse monoclonal Ab (Santa Cruz, clone: 190b, Cat. # sc-13596), anti-human HIF-3α rabbit polyclonal Ab (Abcam, Cat. #ab10134), and anti-human β-actin mouse monoclonal antibody (Santa Cruz, clone: AC-15, Cat. #Sc-69879) to analyze the knockdown efficiency. Data represent means ± SD (n = 3). *P < 0.05, ***P < 0.001.

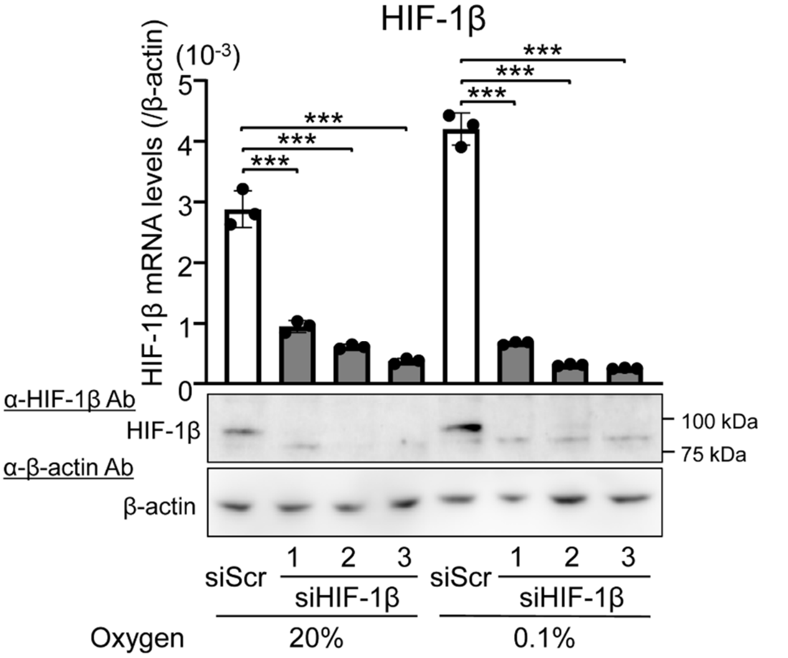

Supplemental Figure 5. Silencing of HIF-1β. After silencing of HIF-1β, HeLa cells were cultured under the indicated oxygen conditions for 24 h, and subjected to qRT-PCR and Western blotting using anti-ARNT/HIF-1β mouse monoclonal Ab (Novus Biologicals, clone: H1beta234, Cat. #NB100-124) and anti-human β-actin mouse monoclonal antibody to analyze the knockdown efficiency. Data represent means ± SD (n = 3). ***P < 0.001.

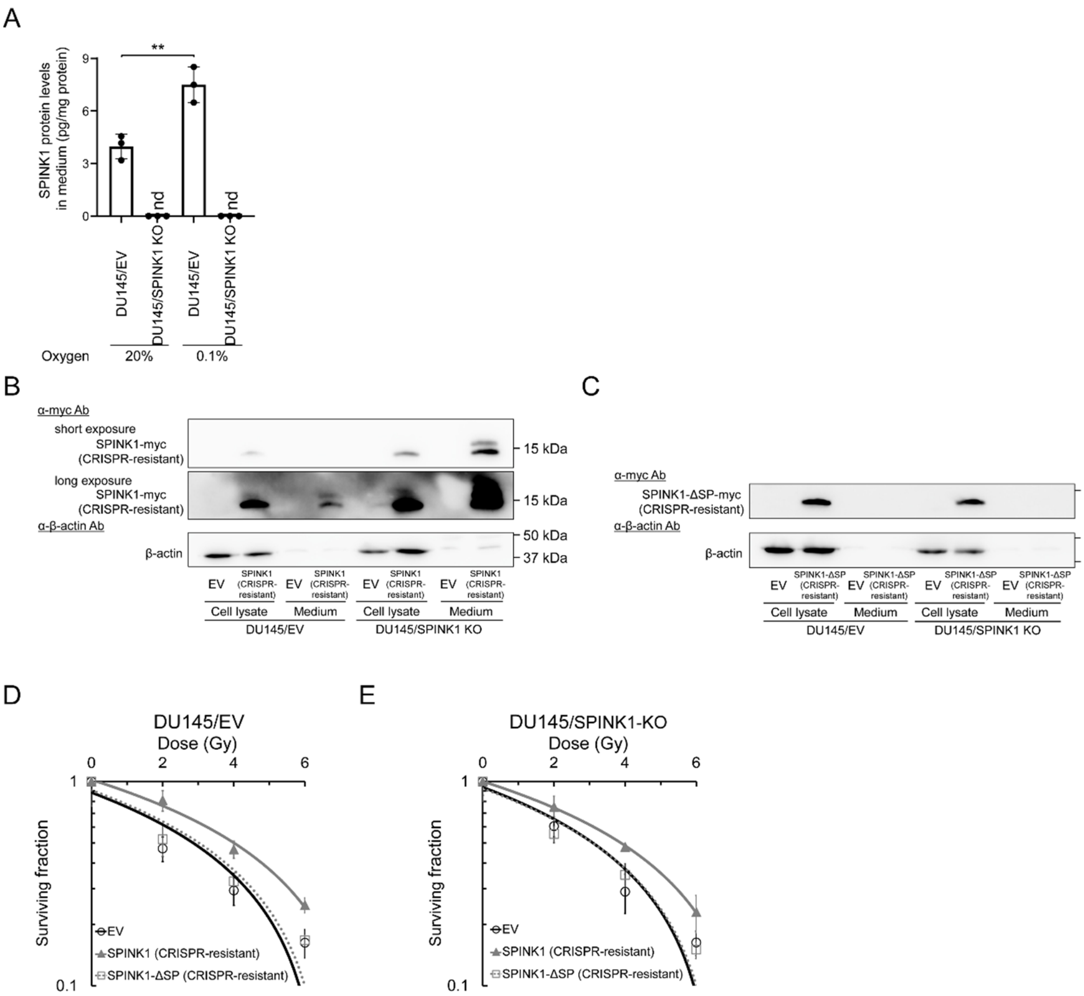

Supplemental Figure 6. Paracrine action of SPINK1 is essential for its activity to induce cellular radioresistance. (**A**) DU145-based SPINK1 KO cells (DU145/SPINK1 KO), which were established by introducing the CRISPR/Cas9-based lentivirus for SPINK1 knockout, or their mock control (DU145/EV) were cultured under the indicated oxygen conditions for 48 hours and subjected to the ELISA assay to confirm SPINK1 knockout. (**B**) The indicated cells were transfected with either empty vector (EV) or the expression vector for SPINK1 (CRISPR-resistant SPINK1), which was wild-type at the protein level but was introduced with mutations at the DNA level and thereby resistant to the CRISPR/Cas9-mediated knockout. The resultant cell lysates and culture media were subjected to Western blotting using anti-myc epitope tag mouse monoclonal antibody (Cell Signaling Technology, clone: 9B11, Cat. #2276) and anti-human β-actin mouse monoclonal antibody (Santa Cruz). The exogenously expressed SPINK1 was detected using anti-myc tag Ab. (**C**) The same experiment as in **B** was performed using the expression vector for CRISPR-resistant SPINK1-ΔSP, which was introduced with an additional mutation lacking the N-terminus signal peptide for secretion. (**D** and **E**) The same experiments as in Figure **1K** and **3K** were performed after transfection with either EV, the expression vector for the CRISPR-resistant SPINK1, or that for the CRISPR-resistant SPINK1-ΔSP into the indicated cells. Data represent means ± SD (n = 3 in **A**; n = 6 in **D** and **E**). ***P* < 0.01. nd, not detected.

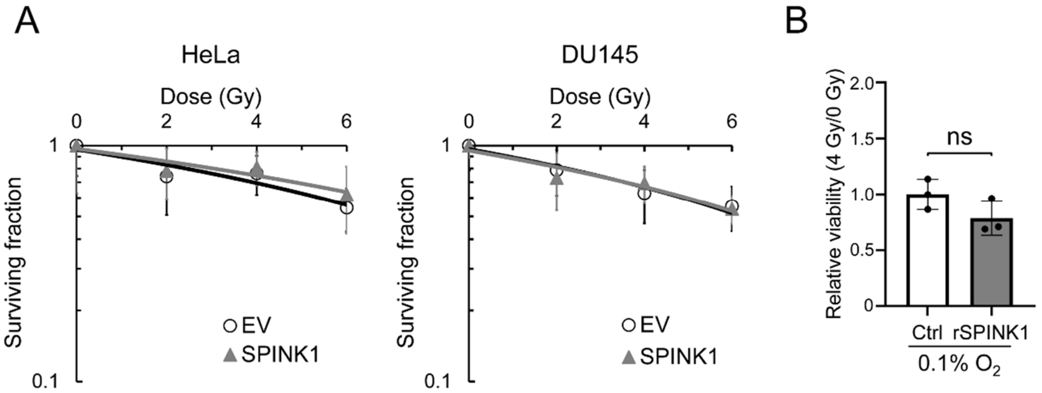

Supplemental Figure 7. SPINK1 does not induce radioresistance of cells under severe hypoxia. (**A**) After transfection with either pcDNA4/SPINK1 (SPINK1) or its empty vector (EV), the indicated cells were precultured under severely hypoxic conditions (O_2 < 0.1%) for 48 h, treated with the indicated dose of γ-ray irradiation under the same conditions as for the preculture, and subjected to clonogenic survival assay. (**B**) Following 24 h serum starvation, DU145 cells were precultured in the presence or absence of 100 ng/mL rSPINK1 for 24 h, treated with the indicated dose of γ-ray irradiation, and subjected to the colorimetric cell viability assay. The cells were precultured and irradiated under severely hypoxic conditions (O_2 < 0.1%). Data represent means ± SD (n = 6 in **A**; n = 3 in **B**). ns, not significant.

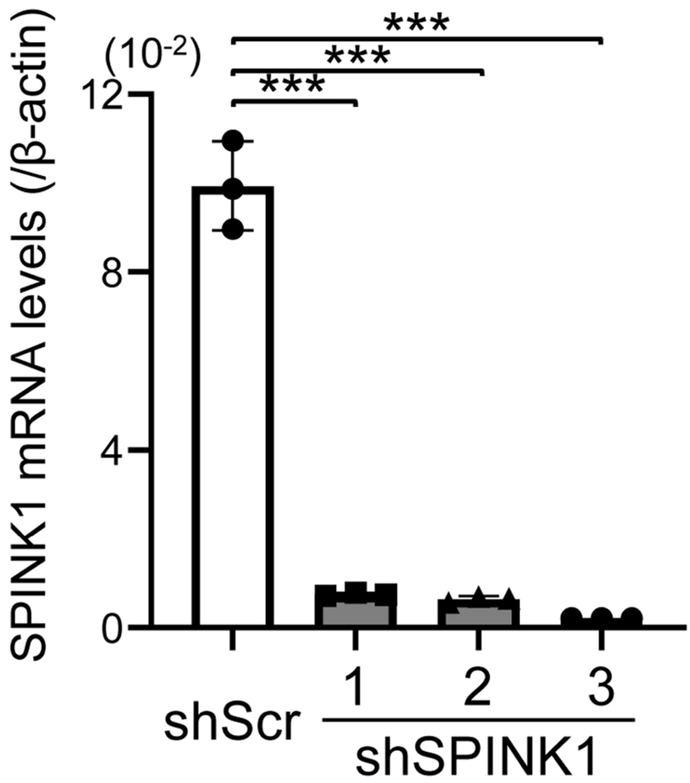

Supplemental Figure 8. Silencing of SPINK1. HeLa/Scr and HeLa/shSPINK1-#1, #2, and #3 were cultured under severely hypoxic conditions (O_2 < 0.1%) for 48 h, and subjected to qRT-PCR to analyze the knockdown efficiency. Data represent means ± SD (n = 3). ****P* < 0.001.

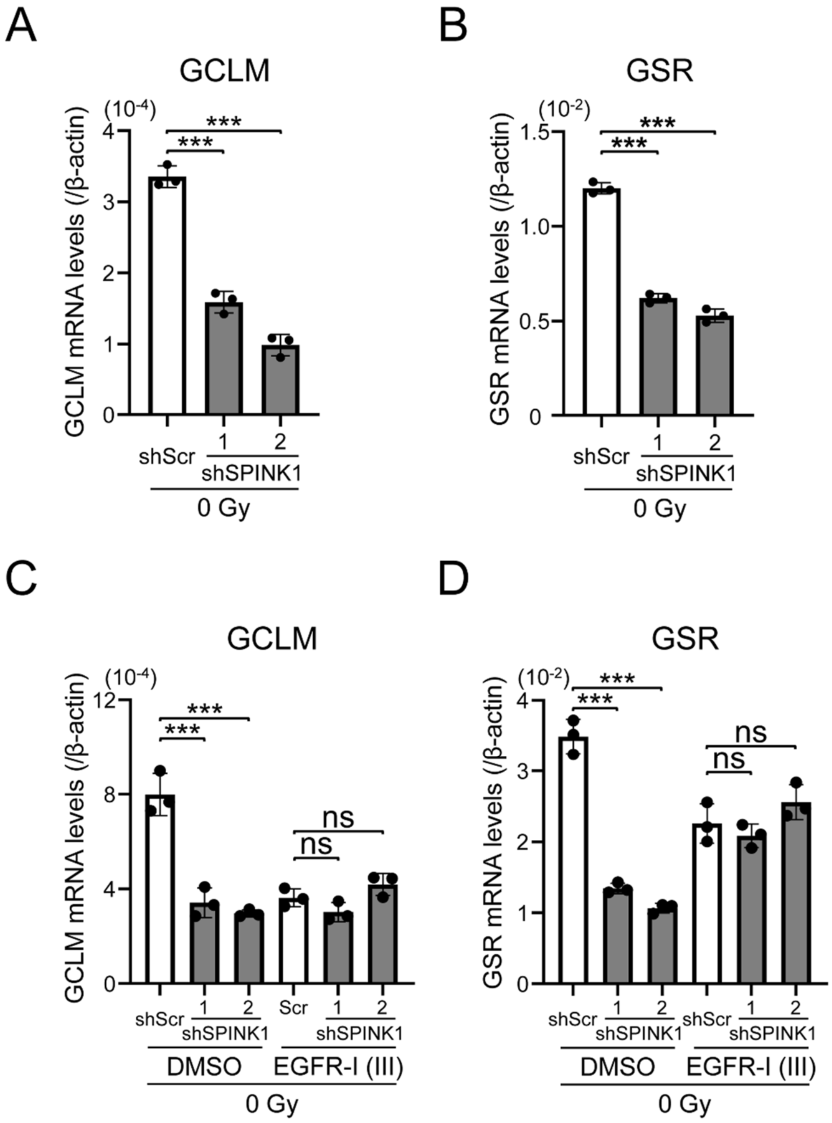

Supplemental Figure 9. Expressions of Nrf2 target antioxidant genes are induced downstream of the SPINK1-EGFR-mediated signaling. The same experiments as in Figure **6E**-**6H** were conducted after 0 Gy of γ-ray irradiation. Data represent means ± SD (n = 3). One-way ANOVA with Dunnett's test. ***P* < 0.01. ****P* < 0.001. ns, not significant.

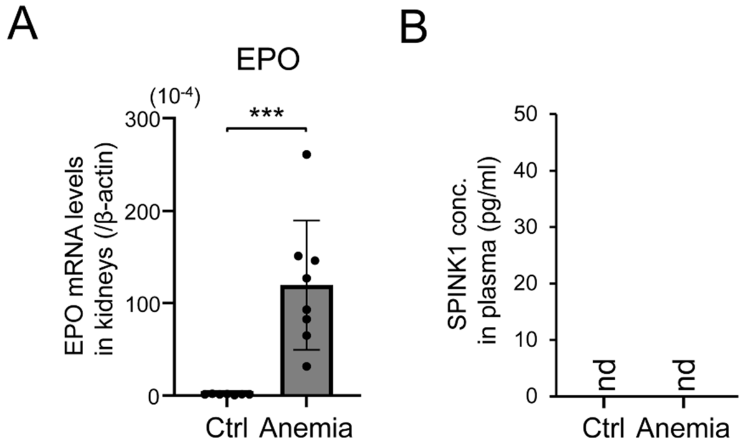

Supplemental Figure 10. Plasma SPINK1 levels are not increased after anemia treatment in non-tumor-bearing mice. (**A** and **B**) After anemia treatment with phenylhydrazine, EPO mRNA levels in kidney (**A**) and SPINK1 protein levels in plasma (**B**) were quantified by qRT-PCR and ELISA assay, respectively. Data represent means ± SD (n = 7 in Ctrl; n = 8 in anemia). ***P < 0.001. nd, not detected.

Supplemental Table 1. Sequence lists of siRNAs used in the present study.

siRNA	Identifier	Sense (5'-3')	Antisense (5'-3')
siHIF-1α (#1)	Cat. #HSS104774	AUAUGAUUGUGUCUCCAGCGGCUGG	CCAGCCGCUGGAGACACAAUCAUAU
siHIF-1α (#2)	Cat. # HSS104775	AGUUAGUUCAAACUGAGUUAAUCCC	GGGAUUAACUCAGUUUGAACUAACU
siHIF-1α (#3)	Cat. #HSS179231	AAGUCUUGCUAUCUAAAGGAAUUUC	GAAAUUCCUUUAGAUAGCAAGACUU
siHIF-2α (#1)	Cat. #HSS103261	GGCCAGGUGAAAGUCUACAACAACU	AGUUGUUGUAGACUUUCACCUGGCC
siHIF-2α (#2)	Cat. #HSS176568	CACCGGCCCAUGUCCUCCAUCUUCU	AGAAGAUGGAGGACAUGGGCCGGUG
siHIF-2α (#3)	Cat. #HSS176569	GGACAAGCCACUGAGCGCAAAUGUA	UACAUUUGCGCUCAGUGGCUUGUCC
siHIF-3α (#1)	Cat. #HSS148869	UCGACCACGGAGCUGCGCAAGGAAA	UUUCCUUGCGCAGCUCCGUGGUCGA
siHIF-3α (#2)	Cat. #HSS184879	GAACCACUGGAUGCCUGCUACCUGA	UCAGGUAGCAGGCAUCCAGUGGUUC
siHIF-3α (#3)	Cat. #10620312	CGGAGAGUAUCGUCUGUGUCCAUUU	AAAUGGACACAGACGAUACUCUCCG
siHIF-1β (#1)	Cat. #HSS100699	GGGAACUGGCAACACAUCCACUGAU	AUCAGUGGAUGUGUUGCCAGUUCCC
siHIF-1β (#2)	Cat. #HSS100700	GCAGCACACUCUAUGAUCAGGUGCA	UGCACCUGAUCAUAGAGUGUGCUGC
siHIF-1β (#3)	Cat. #HSS100701	GGAAGGUCAGCAGUCUUCCAUGAGA	UCUCAUGGAAGACUGCUGACCUUCC

Supplemental Table 2. Targeting sequences of shRNA and sgRNA vectors.

shRNA	Identifier (Barcode ID)	Target	Targeting Sequence : Sense (5'-3')	Targeting Sequence : antisense (5'-3')
shSPINK1 (#1)	12214	SPINK1 CDS	GCCAAATGTTACAATGAACTT	AAGTTCATTGTAACATTTGGC
shSPINK1 (#2)	12216	SPINK1 CDS	CCCTGTTGAGTCTATCTGGTA	TACCAGATAGACTCAACAGGG
shSPINK1 (#3)	12218	SPINK1 CDS	TGATGGAAATACTTATCCCAA	TTGGGATAAGTATTTCCATCA
sgSPINK1	N/A (original)	SPINK1 CDS	ACAGACAGGGTCATATATCT	AGATATATGACCCTGTCTGT

Supplemental Table 3. List of primers used in qRT-PCR experiments.

Target gene	Forward primer (5'-3')	Reverse primer (5'-3')
ACTB	TGGCACCCAGCACAATGAA	CTAAGTCATAGTCCGCCTAGAAGC
SPINK1	CCTTGGCCCTGTTGAGTCTA	TGTAACATTTGGCCTCTCTTCC
CA9	ACCAGACAGTGATGCTGAGTGCTAA	TCAGCTGTAGCCGAGAGTCACC
VEGFA	TCACAGGTACAGGGATGAGGACAC	CAAAGCACAGCAATGTCCTGAAG
HIF-1α	TCATCCAAGAAGCCCTAACGTG	TTTCGCTTTCTCTGAGCATTCTG
HIF-2α（EPAS1)	CACTGCAGACTTGTCCAGTGCTC	CACTGCTCGGATTGTCACACCTA
HIF-3α	ACGGAGCGGTGCTTCTCCTTG	AGTCTGCGCAGGTGGCTTGT
HIF-1β (ARNT)	CTACCCGCTCAGGCTTTTC	CACCAAACTGGGAAGTACGAG
GCLM	AAGCACTTTCTCGGCTACGA	GCGGGAGAGCTGATTCCAAA
GSR	AACAACATCCCAACTGTGGTC	TCCATATTTATGAATGGCTTCATCT
ACTB (mouse)	CATCCGTAAAGACCTCTATCCCAAC	ATGGAGCCACCGATCCACA
EPO (mouse)	TCTGCGACAGTCGAGTTCTG	CTTCTGCACAACCCATCGT

Supplemental Table 4. D_{50} values (The dose of radiation needed to reduce the number of surviving colonies by 50%) in clonogenic survival assays.

Figure	Cell	Oxygen condition	Treatment	D_{50} Value (Gy)	*P* value	Enhancement ratio
Supplemental Figure 6D	DU145/EV	20%	*1pcDNA4/EV	2.87 ± 0.08		
			*1pcDNA4/SPINK1 (CRISPR-resistant)	4.00 ± 0.37	0.0067 (vs pcDNA4/EV)	1.3937 (vs pcDNA4/EV)
			*1pcDNA4/SPINK1-ΔSP (CRISPR-resistant)	2.91 ± 0.10	0.5991 (vs pcDNA4/EV)	1.0139 (vs pcDNA4/EV)
Supplemental Figure 6E	DU145/SPINK1 KO	20%	*1pcDNA4/EV	2.95 ± 0.21		
			*1pcDNA4/SPINK1 (CRISPR-resistant)	3.72 ± 0.15	0.0068 (vs pcDNA4/EV)	1.2610 (vs pcDNA4/EV)
			*1pcDNA4/SPINK1-ΔSP (CRISPR-resistant)	3.08 ± 0.10	0.4118 (vs pcDNA4/EV)	1.0441 (vs pcDNA4/EV)
Supplemental Figure 7A	HeLa	< 0.1%	**1pcDNA4/EV	5.41 ± 1.60	0.6843	1.1220
			*1pcDNA4/SPINK1	6.07 ± 2.05		
	DU145	< 0.1%	*1pcDNA4/EV	5.01 ± 1.07	0.3547	1.1597
			*1pcDNA4/SPINK1	5.81 ± 0.77		

*1: transient transfection with.
